# Gut microbiome insights from 16S rRNA analysis of 17-year periodical cicadas (*Hemiptera*: *Magicicada* spp.) Broods II, VI, and X

**DOI:** 10.1038/s41598-022-20527-7

**Published:** 2022-10-10

**Authors:** Kyle D. Brumfield, Michael J. Raupp, Diler Haji, Chris Simon, Joerg Graf, John R. Cooley, Susan T. Janton, Russell C. Meister, Anwar Huq, Rita R. Colwell, Nur A. Hasan

**Affiliations:** 1 Maryland Pathogen Research Institute, Department of Cell Biology and Molecular Genetics, University of Maryland, College Park, MD 20742 USA; 2 University of Maryland Institute for Advanced Computer Studies, University of Maryland, College Park, MD 20742 USA; 3 Department of Entomology, University of Maryland, College Park, MD 20742 USA; 4 Department of Ecology and Evolutionary Biology, University of Connecticut, Storrs, CT 06269 USA; 5 Department of Integrative Biology, University of California, Berkeley, CA 94720 USA; 6 Department of Molecular and Cell Biology, University of Connecticut, Storrs, CT 06269 USA; 7 Department of Ecology and Evolutionary Biology, University of Connecticut, Hartford, CT 06103 USA; 8 EZbiome Inc., Gaithersburg, MD 20878 USA

**Keywords:** Biodiversity, Metagenomics

## Abstract

Periodical cicadas (*Hemiptera*: *Magicicada*) have coevolved with obligate bacteriome-inhabiting microbial symbionts, yet little is known about gut microbial symbiont composition or differences in composition among allochronic *Magicicada* broods (year classes) which emerge parapatrically or allopatrically in the eastern United States. Here, 16S rRNA amplicon sequencing was performed to determine gut bacterial community profiles of three periodical broods, including II (Connecticut and Virginia, 2013), VI (North Carolina, 2017), and X (Maryland, 2021, and an early emerging nymph collected in Ohio, 2017). Results showed similarities among all nymphal gut microbiomes and between morphologically distinct 17-year *Magicicada*, namely *Magicicada septendecim* (Broods II and VI) and 17-year *Magicicada cassini* (Brood X) providing evidence of a core microbiome, distinct from the microbiome of burrow soil inhabited by the nymphs. Generally, phyla *Bacteroidetes* [*Bacteroidota*] (> 50% relative abundance), *Actinobacteria* [*Actinomycetota*], or *Proteobacteria* [*Pseudomonadota*] represented the core. *Acidobacteria* and genera *Cupriavidus*, *Mesorhizobium*, and *Delftia* were prevalent in nymphs but less frequent in adults. The primary obligate endosymbiont, *Sulcia* (*Bacteroidetes*), was dominant amongst core genera detected. *Chryseobacterium* were common in Broods VI and X. *Chitinophaga, Arthrobacter*, and *Renibacterium* were common in Brood X, and *Pedobacter* were common to nymphs of Broods II and VI. Further taxonomic assignment of unclassified *Alphaproteobacteria* sequencing reads allowed for detection of multiple copies of the *Hodgkinia* 16S rRNA gene, distinguishable as separate operational taxonomic units present simultaneously. As major emergences of the broods examined here occur at 17-year intervals, this study will provide a valuable comparative baseline in this era of a changing climate.

## Introduction

Diverse microbial communities flourish in a wide spectrum of complex environments ranging from the rhizosphere of plants, the gut of humans and other eukaryotes, and even in conventionally inhospitable habitats. These microbes play critical roles in the biogeochemistry of the planet and in maintaining life globally^1^. A prime example of these complex microbial communities is found in insects, which comprise *ca.* 90% of all known animal species. Nearly all insect species are associated with endosymbiotic bacteria, many of which are able to form mutualistic relationships and/or influence biological functions of their insect host^2^. While some important endosymbiotic microbiota can also be selected from the environment by their insect hosts, others are inherited from the parent. Inherited microbes are for the most part obligate such that insects lacking their bacteria are unable to develop properly, and their bacteria are unable to reproduce outside the host^2,3^. Obligate endosymbiotic microbiota provide their hosts with nutritional compounds essential for survival and development^4^. They are generally found in specialized cells (bacteriocytes) contained within the bacteriome that provide nutrients to the bacteria^4–7^.

Cicadas, members of superfamily *Cicadoidea*, order *Hemiptera*, are plant sucking insects, adults of which range in body length from one to seven cm, and are commonly found in diverse environments, including deserts, grasslands, and forests (Fig. S1). More than 3000 cicada species have been described^8^. Periodical cicadas of the genus *Magicicada* emerge in North America as locally synchronized populations with a common emergence schedule at 13- or 17-year intervals, and are among the highest reported biomasses of all naturally occurring terrestrial animals, with average emergence densities^9^ of up to 600 cicadas/m^2^. Periodical cicadas are divided into seven species, two life cycles, and 15 allochronic broods that are largely allopatric or parapatric^10^. The current molecular phylogeny recognizes four reproductively isolated lineages. With the exception of the *Magicicada tredecim* lineage*,* lifecycles within the morphologically distinct species lineages hybridize^11–13^.

**Figure 1:**
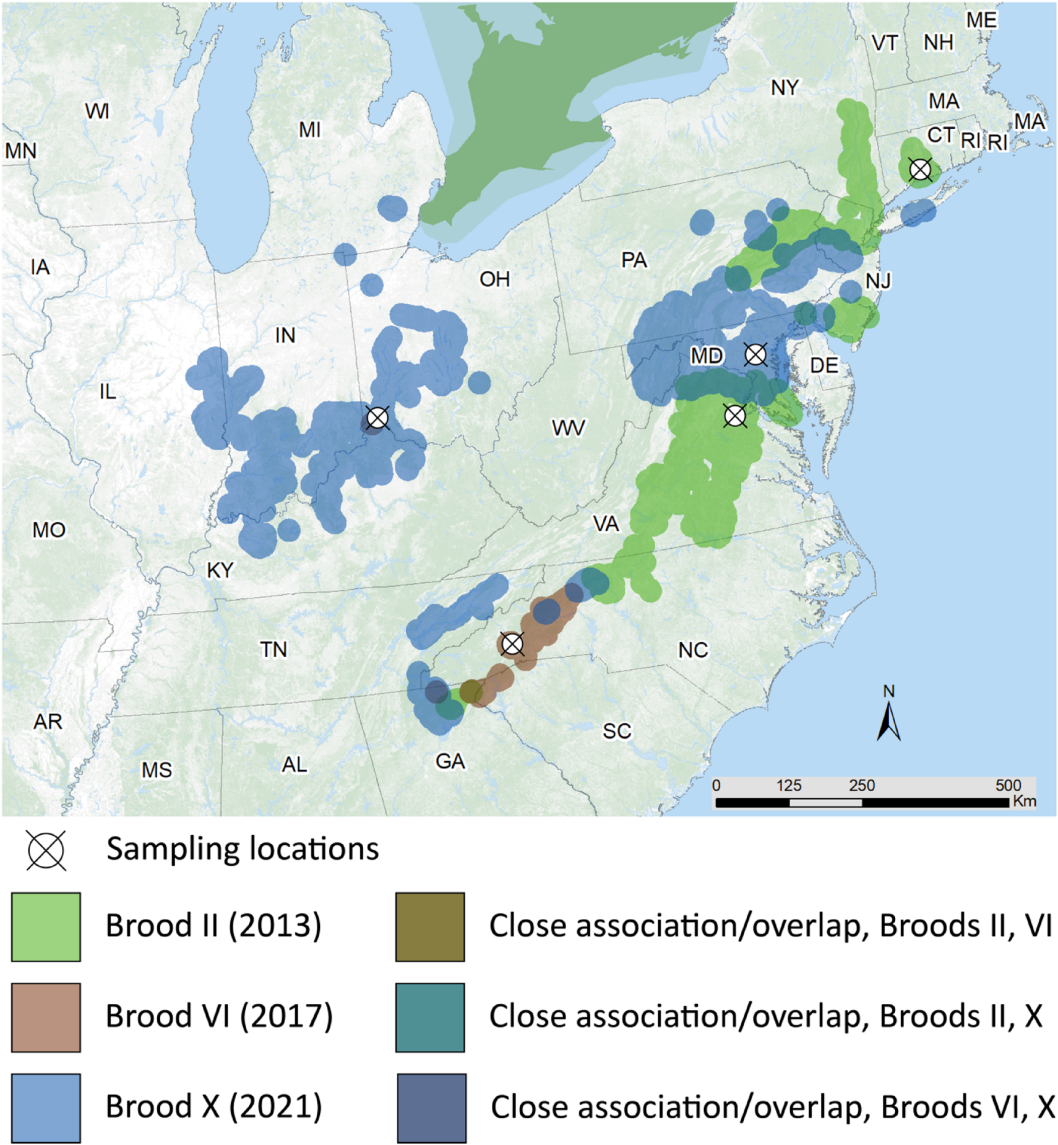
Map of brood emergences and sampling locations. Shown are regions where and when different broods of periodical cicadas (Broods II, VI, and X) are likely to emerge. Map was created using ARCgis Online (Environmental Systems Research Institute)^68^. Cicada brood emergence was compiled by Simon and colleagues^12^.

Twelve extant broods of 17-year and three of 13-year cicadas have been described and appear regularly throughout the eastern U.S.^12^. Most broods contain three morphologically distinct species, exhibiting significant overlap between broods^14^. This paper focuses on cicadas sampled from three 17-year cicada broods: Broods II, VI, and X. These three broods are separated in time by at least 4 years and overlap in mosaic fashion where their ranges intersect. Brood II emerges primarily along the U.S. eastern seaboard, but disjunct populations in in Oklahoma and northeast Georgia and have been reported^15^. Brood VI core populations emerge in North Carolina, South Carolina, and Georgia^12,16^. Brood X, considered among the largest broods by geographic extent of 17 year cicadas, emerged most recently, beginning in April 2021. Emergence increased during the first weeks of May 2021 with large numbers observed in Georgia, Tennessee, Maryland, and Washington, D.C., and as the season progressed, vast numbers emerged in more than a dozen states in the eastern half of the U.S.^17^. However, a changing climate with longer periods of warmth during which the underground nymphs can grow could be triggering cicadas to emerge ahead of their brood^18,19^. For example, it has been suggested that the schedule of Brood X, which is closely associated with Broods VI and XIV, may be changing because in many local populations noticeable numbers of individuals emerged four years early in 2017, some even numerous enough to chorus and lay eggs^12,20^. A map depicting sampled periodical cicada brood distributions and expected years of emergence (Broods II, VI, and X) is shown in Fig. 1.

Like other cicadas, *Magicicada* feed on xylem fluid of roots, stems, and branches^21^, which is a nutrient-poor food source, providing carbohydrates, namely sucrose, but poor in nitrogenous compounds, e.g., amino acids and vitamins^12,22^ and have coevolved with the obligate bacteriome-dwelling co-partner endosymbionts *Sulcia muelleri* (hereafter, *Sulcia*) and *Hodgkinia cicadicola* (hereafter, *Hodgkinia*)^6,12,23–27^. While it is clear that cicadas are dependent on their specialized bacteriome-dwelling endosymbionts, the composition of endosymbiont communities dwelling outside of the bacteriome has been assumed to be unimportant and only recently begun to be explored^5–7,24,25,28–31^. However, comparative analysis of *Magicicada *spp. gut microbiomes from different broods has yet to be done.

This study is the first to use metagenomic 16S rRNA amplicon sequencing to identify microbiome components of *Magicicada *spp*.* nymphs, adults, and the soil they inhabit. We profile the gut microbiome of multiple individuals of 17-year cicadas (Broods II, VI, and X). We find that cicadas do have a core microbiome different from the soil they inhabit. We compare and contrast gut-endosymbiont composition among individuals, broods, and sexes. Results provide an initial survey of bacterial taxa comprising the core microbiome, along with taxonomic biomarkers. As major emergences of the broods examined here occur at 17-year intervals, this study will provide a valuable comparative baseline in this era of a changing climate.

## Results

### Metagenomic 16S rRNA amplicon sequencing

Metagenomic 16S rRNA amplicon sequencing, using DNA prepared from the complete gut, filter chamber, midgut, Malpighian tubules and hindgut, and rectum (Fig. S2) generated *ca.* 6.40 × 10^6^ reads across the raw sequence libraries (mean = 1.02 × 10^5^ reads per sample). Following taxonomic profiling of all microbiome samples, sparsity was calculated to be 92%, with 3.7% of the operational taxonomic units (OTUs; defined using 97% similarity boundary) profiled as singletons. The proportion of classified reads varied between cohorts, with cicada samples (min = 2.93%, A2; max = 99.7%, N4_M; mean = 59.9%) showing a greater abundance of unclassified reads than soil samples (mean = 92.8%). The relative abundance (RA) of reads classified as bacteria relative to the total number of reads generated in each sample is shown in Fig. S3.

### Alpha diversity

**Figure 2:**
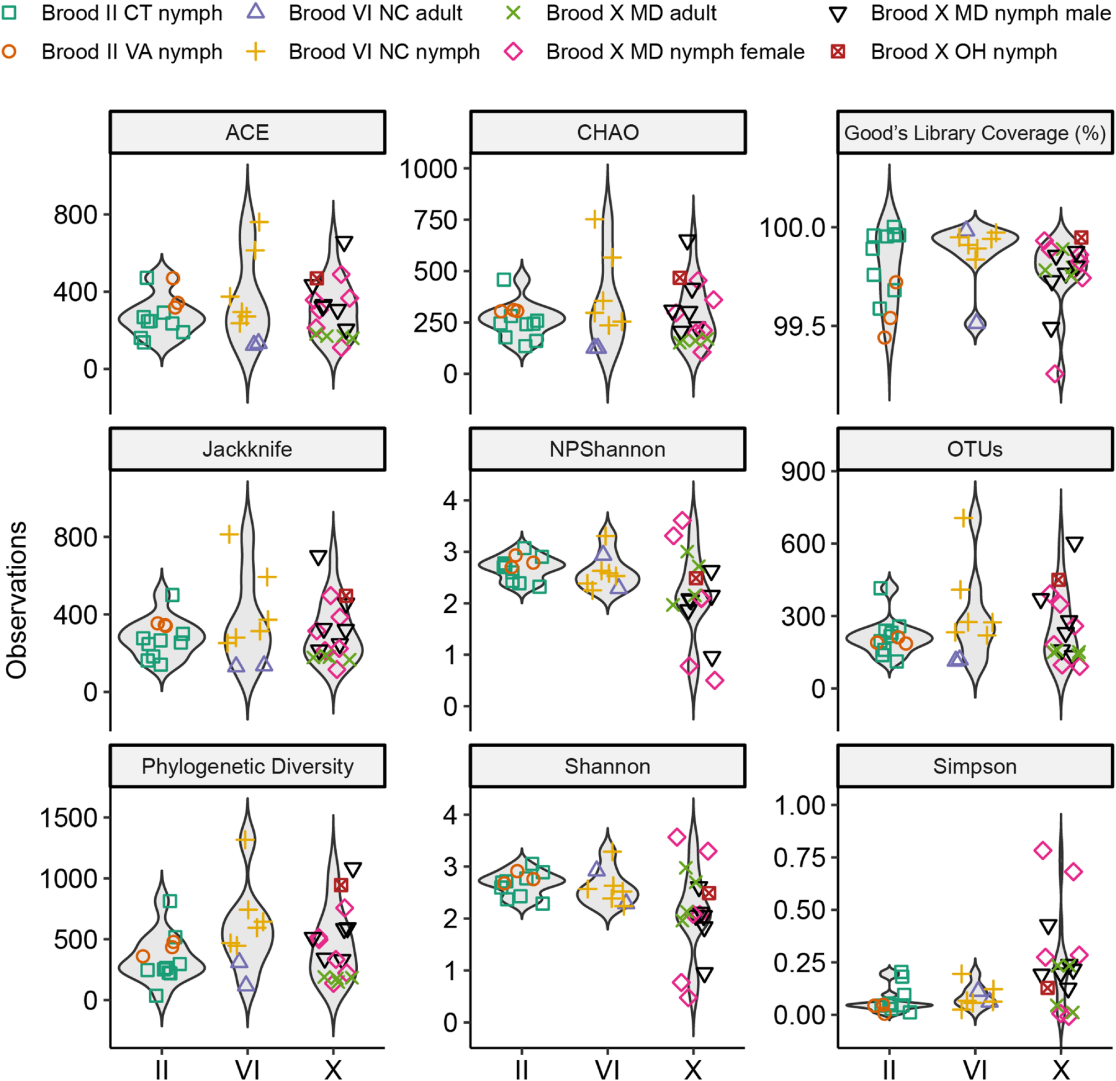
Violin plots showing alpha diversity comparison between MTP sets of cicada samples.

Total bacterial alpha diversity was calculated between cicada microbiome taxonomic profiling (MTP) sets, i.e., Brood II CT nymphs, Brood II VA nymphs, Brood VI NC adults, Brood VI NC nymphs, Brood X MD adults, Brood X MD female nymphs, Brood X MD male nymphs, and the Brood X OH nymph, using metrics for species richness (ACE, Chao1, Jackknife, and number of OTUs in each MTP set), diversity index (NPShannon, Shannon, Simpson, and phylogenetic diversity), and Good’s coverage (Fig. 2). While the alpha diversity of soil samples was significantly higher than detected in cicada samples (Fig. S4), only minor differences were observed between MTP sets, with Brood X adult cicadas showing the least variability across samples within the same MTP set. In general, alpha diversity of nymphs was slightly greater than adults (Brood VI and X), and Brood X male nymphs was slightly higher than detected amongst Brood X female nymphs. However, no significant differences of alpha diversity between sampling times, location, brood, or life stage were observed following Wilcoxon rank-sum test. Hence, the overall alpha diversity of cicadas between MTP sets are concluded to be similar.

### Beta diversity

**Figure 3:**
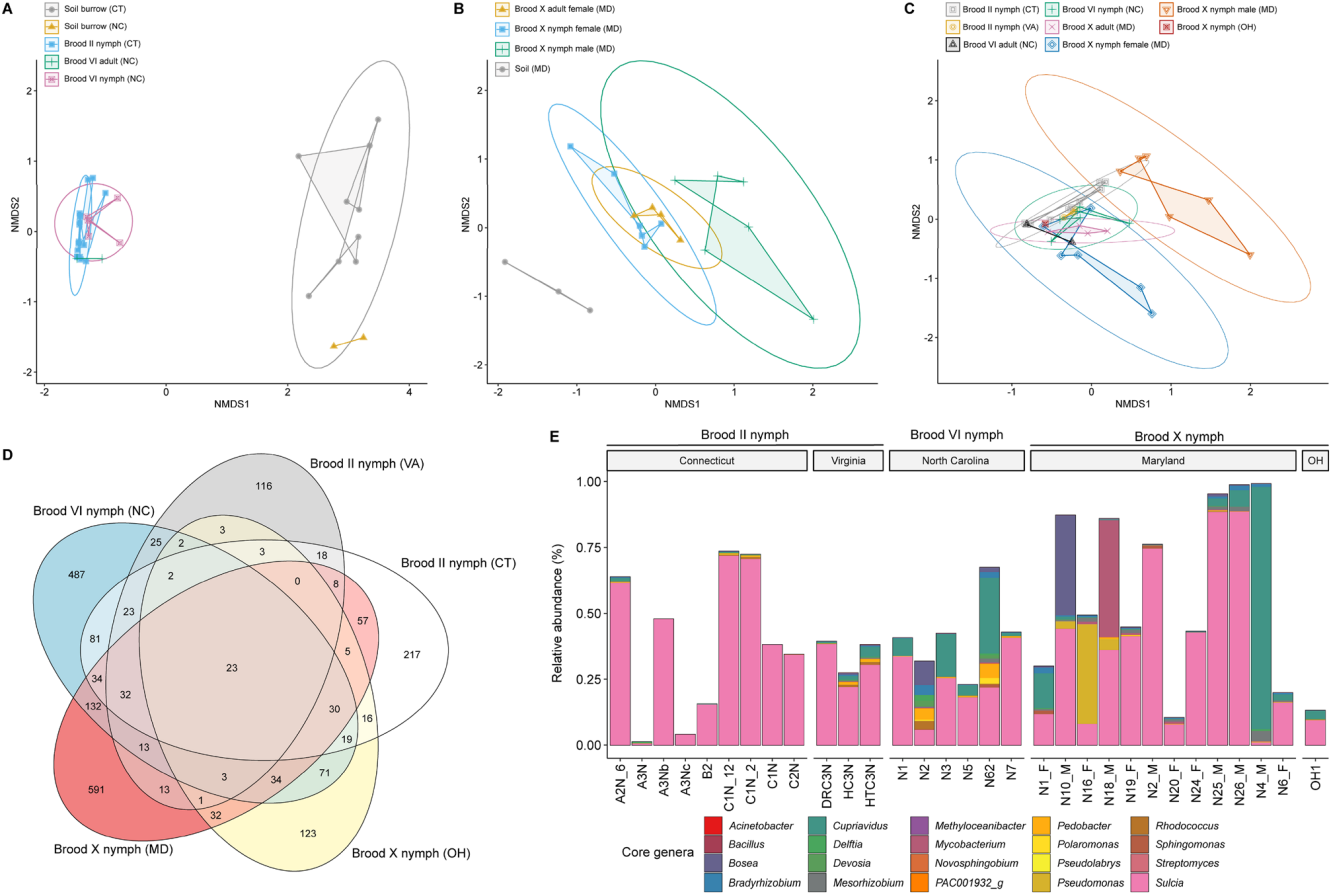
Beta diversity indices. NMDS plot of nymph gut microbiomes and microbiomes of (**A**) soil inhabited by nymphs in CT (Brood II) and NC (Brood VI), (**B**) Brood X gut microbiomes and microbiomes of soil in MD, and (**C**) cicada gut microbiomes showing life stage, brood, collection location, and sex of Brood X cicadas. Ellipses represents 95% confidence interval based on Bray–Curtis dissimilarity index. (**D**) Venn diagram of bacterial communities showing the number of shared and exclusive bacterial taxa is shown relative to MTP set. (**E**) Relative abundance of 20 most common core bacterial genera detected across all MTP sets.

Bacterial community profiles for the cicada samples were analyzed by non-metric multidimensional scaling (NMDS) using Bray–Curtis dissimilarity index (Fig. 3), where distance between points indicates dissimilarities in bacterial DNA sequence composition. Overall, soil samples formed distinct clusters (Fig. 3), with respect to CT Brood II nymphs and both nymphs and adults in NC (Brood VI) and MD (Brood X). Interestingly, Brood X female nymphs and female adults clustered more closely together with other cicada MTP sets, compared to Brood X male nymphs (Fig. 3C). The Venn diagram (Fig. 3D) represents profiles for the bacteria, with respect to number of shared and exclusive bacterial taxa, i.e., unique bacterial taxa detected in a set of samples and not detected in other samples across MTP sets, detected in nymph gut microbiomes. The number of exclusive taxa was largest for MD Brood X nymphs (591 taxa) followed by Brood VI nymphs (487 taxa), compared to Brood II nymphs which contained fewer exclusive taxa (CT, 217 taxa; VA, 116 taxa). MD Brood X and Brood VI nymphs shared more taxa in common between MTP sets than with other broods. A core microbiome, i.e., taxa detected in all samples, was compiled for all the MTP sets, with 23 common taxa detected. Figure 3E shows the RA of the 20 most common core bacterial genera detected across all MTP sets. The core microbiome of all samples was dominated by *Sulcia*, but *Cupriavidus* was also detected frequently.

Table 1 shows results of Beta set-significance analysis employing Bray–Curtis dissimilarity index. Permutational multivariate analysis of variance (PERMANOVA) results indicated that the gut microbiomes of nymphs and the soil they inhabit are significantly different (q < 0.05). Minor differences were observed between Brood II and Brood VI nymphs (P < 0.05) but statistical comparisons with Brood X could not be done due to differences in sequencing method. Within the MD Brood X samples, male nymphs contained a distinct bacterial community, compared to female nymphs (q = 0.034) and female adults (q = 0.039), while the bacterial microbiomes of female nymphs and female adults were more closely related (not significant). Lastly, it is worth noting that the gut microbiomes between Brood II nymph populations in CT and VA were strikingly similar in favor of H_0_ (Pseudo-F = 0.37).

**Table 1:** Beta diversity set-significance analysis. Permutational multivariate analysis of variance (PERMANOVA) results are shown between MTP sets employing Bray–Curtis Beta diversity distance measure and corrected for multiple comparisons following read and gene copy number normalization.

H_0_: No difference between MTP sets	Pseudo-F	P value	Q value^a^	Accept/reject H_0_
**Soil microbiomes**				
Soil without nymphs (CT) = soil inhabited by nymphs (CT)	0.853	0.478	0.561	–
Soil inhabited by nymphs (CT) = soil inhabited by nymphs (NC)	2.676	*(0.015)	0.068	–
**Nymph gut microbiomes and soil microbiomes**				
Brood II nymphs (CT) = soil inhabited by nymphs (CT)	21.799	***(0.001)	*(0.014)	Reject
Brood VI nymphs (NC) = soil inhabited by nymphs (NC)	12.536	*(0.043)	0.1161	–
Nymphs (CT + NC) = soil inhabited by nymphs (CT + NC)	28.283	***(0.001)	*(0.014)	Reject
Brood X nymphs (MD) = soil without nymphs (MD)	3.095	**(0.002)	*(0.018)	Reject
**Adult gut microbiomes and soil microbiomes**				
Brood VI adults (NC) = soil inhabited by nymphs (NC)	4.470	0.333	0.450	–
Brood X adults (MD) = soil without nymphs (MD)	5.395	*(0.033)	0.116	–
**Microbiomes of Brood II (CT) gut dissects**				
Midgut = other dissects^b^	0.067	0.774	0.804	–
Midgut = whole gut	1.591	0.203	0.365	–
Other dissects^b^ = whole gut	1.588	0.231	0.367	–
Midgut and other dissects^b^ = whole gut	2.768	0.081	0.199	–
**Nymph gut microbiomes**				
Brood II nymphs (CT) = Brood II nymphs (VA)	0.431	0.65	0.702	–
Brood II nymphs (CT) = Brood VI nymphs (NC)	1.887	0.115	0.239	–
Brood II nymphs (VA) = Brood VI nymphs (NC)	0.930	0.435	0.559	–
Brood II nymphs (CT + VA) = Brood VI nymphs (NC)	3.987	*(0.043)	0.116	–
Brood X male nymphs (MD) = Brood X female nymphs (MD)	3.301	**(0.005)	*(0.034)	Reject
**Nymph and adult gut microbiomes**				
Brood VI nymphs (NC) = Brood VI adults (NC)	1.723	0.2	0.365	–
Brood X male nymphs (MD) = Brood X female adults (MD)	3.739	**(0.007)	*(0.039)	Reject
Brood X female nymphs (MD) = Brood X female adults (MD)	1.569	0.219	0.367	–
***Hodgkinia* OTUs in nymph gut microbiomes**				
Brood II nymphs (CT) = Brood II nymphs (VA)	0.3676	0.999	0.999	–
Brood II nymphs (CT) = Brood VI nymphs (NC)	0.847	0.604	0.680	–
Brood II nymphs (VA) = Brood VI nymphs (NC)	2.059	0.109	0.239	–
Brood II nymphs (CT + VA) = Brood VI nymphs (NC)	2.557	*(0.037)	0.116	–
Brood X male nymphs (MD) = Brood X female nymphs (MD)	2.059	0.315	0.448	–
***Hodgkinia* OTUs in Nymph and adult gut microbiomes**				
Brood VI nymphs (NC) = Brood VI adults (NC)	1.714	0.262	0.393	–
Brood X nymphs (MD) = Brood X adults (MD)	0.949	0.473	0.561	–

^a^Q value, False Discovery Rate (FDR) adjusted P value; ^b^other dissects include filter chamber (n = 1), Malpighian tubules and hindgut (n = 2), and rectum (n = 1); (*) P ≤ 0.05, (**) P ≤ 0.01, (***) P ≤ 0.001; (–) accept H_0_.

### Taxonomic composition

**Figure 4:**
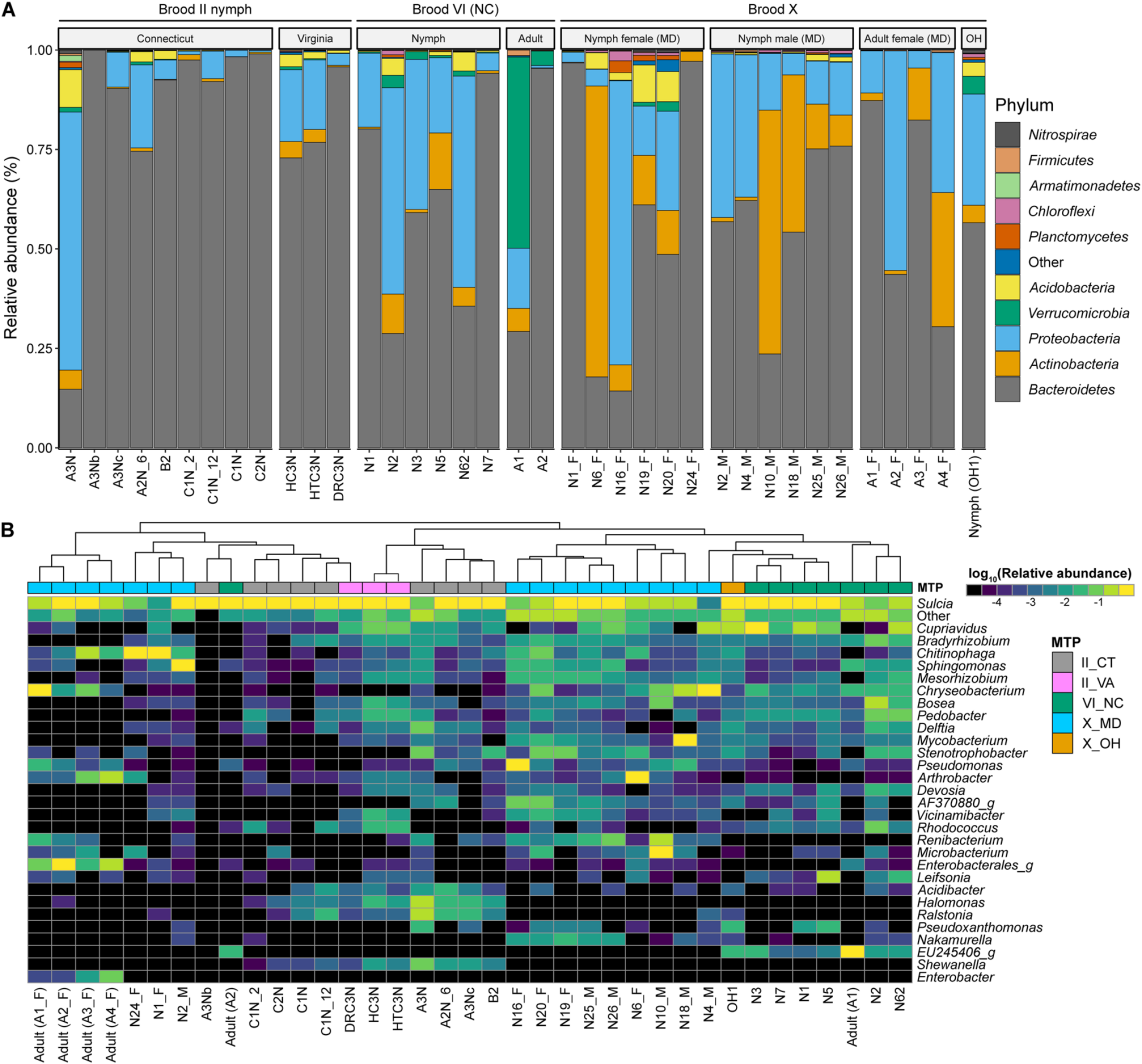
Bacterial community composition. (**A**) Stacked bar plot showing relative sequencing read abundance of 10 most abundant bacterial phyla. (**B**) Heatmap showing log_10_(relative abundance) of 30 most abundant bacterial genera. Dendrogram shows k-means clustering of samples.

Overall, individuals within MTP sets varied in RA of dominant phyla detected, with greatest diversity at the phylum level for MD Brood X female nymphs, followed by Brood VI nymphs, compared to MD Brood X male nymphs and cicada adults, for which fewer phyla were detected (Fig. 4A). Across all MTP sets, *Bacteroidetes* [*Bacteroidota*], *Actinobacteria* [*Actinomycetota*], and *Proteobacteria* [*Pseudomonadota*] were dominant. *Chloroflexi* was detected in most of the MD Brood X nymph samples (both male and female), and also in the OH Brood X nymph and a few of the Brood VI nymphs. Similarly, *Planctomycetes*, *Verrucomicrobia*, and *Acidobacteria* were detected at increased abundance in a few of the samples.

Hierarchical cluster analysis suggests that the community structure differed between broods at the genus level, as samples generally clustered with like samples from the same brood, respectively (Fig. 4B). Similarly, the profiles of Brood X female adults were clearly different from those of Brood X nymphs. *Sulcia* was detected in all samples. The genera *Cupriavidus, Chitinophaga*, *Sphingomonas*, and *Chryseobacterium* were detected in most samples, regardless of brood or life stage. Bacteria commonly associated with soil, e.g., *Mesorhizobium* and *Bradyrhizobium*, were common in nymphs but not adult cicadas. *Shewanella*, *Halomonas*, and *Ralstonia* were common to Brood II nymphs, *Nakamurella* was common between Brood VI and MD Brood X nymphs, and *Enterobacter* was detected frequently among the MD Brood X adults.

### *Magicicada *spp*.* core microbiome

**Figure 5:**
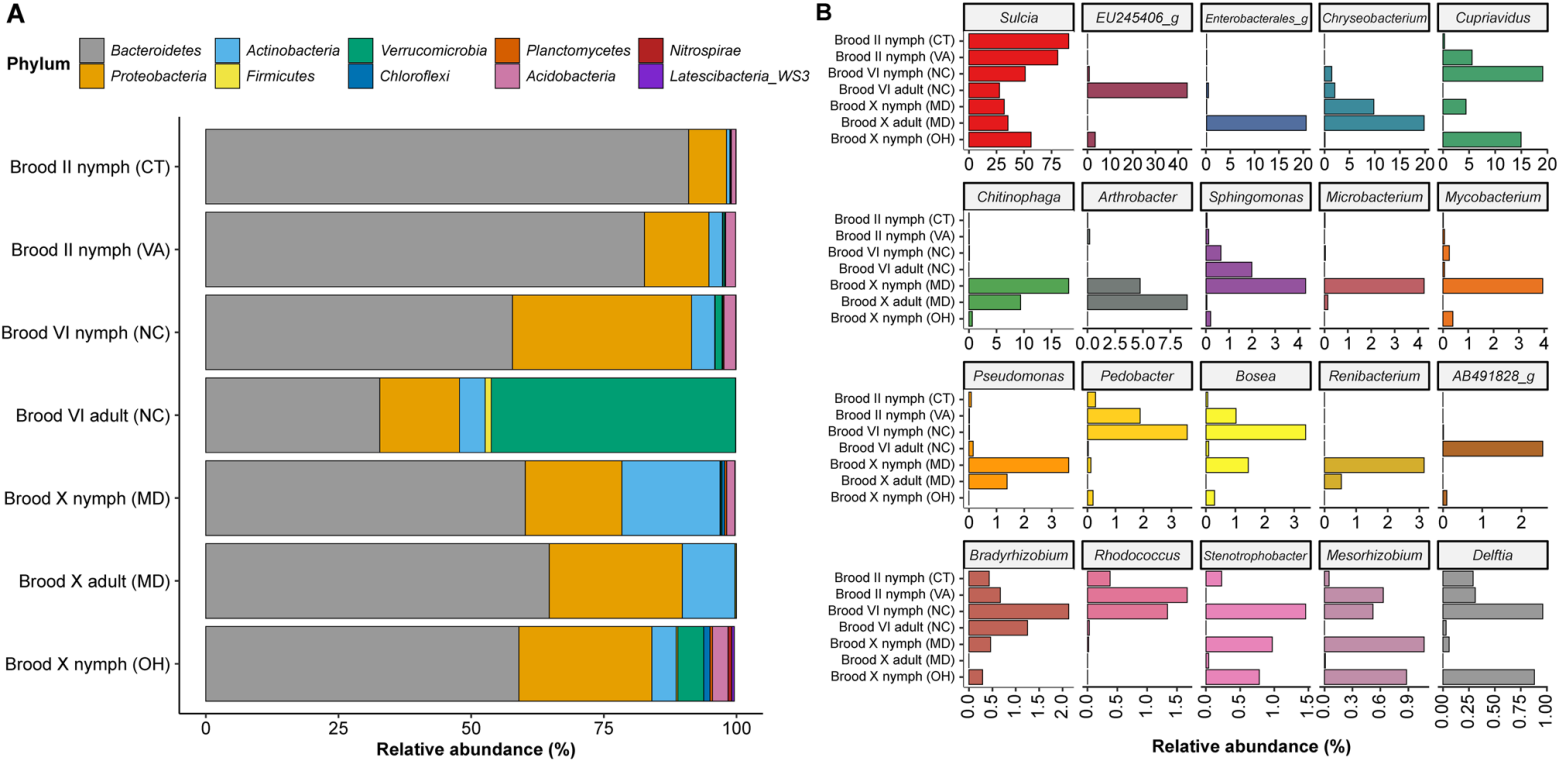
Average bacterial community compositions. Stacked bar plot showing relative abundance of (**A**) ten most abundant bacterial phyla detected across MTP set and (**B**) twenty most abundant bacterial genera detected across MTP set, ordered from most abundant to least abundant.

The core microbiome of *Magicicada *spp. was profiled by evaluating the average taxonomic abundance between MTP sets at the phylum (Fig. 5A) and genus (Fig. 5B) taxonomic rankings. Generally, the phyla *Bacteroidetes* (> 50% RA), *Actinobacteria* (> 10% RA), or *Proteobacteria* (> 10%) represented the core. *Acidobacteria* were prevalent in nymphs but detected less frequently in adults. At the genus level, *Sulcia* were the most abundant of the core bacterial taxa detected. However, *Chryseobacterium* was common in MD Brood X and NC Brood VI cicadas, including nymphs and adults. *Cupriavidus*, *Mesorhizobium*, and *Delftia* were prevalent in nymphs but less frequent in adults. *Chitinophaga, Arthrobacter*, and *Renibacterium* were common in MD Brood X samples (nymphs and adults), while *Pedobacter* was common to Brood II and Brood VI nymphs.

### Microbiome community composition differentiated by MTP set

**Figure 6:**
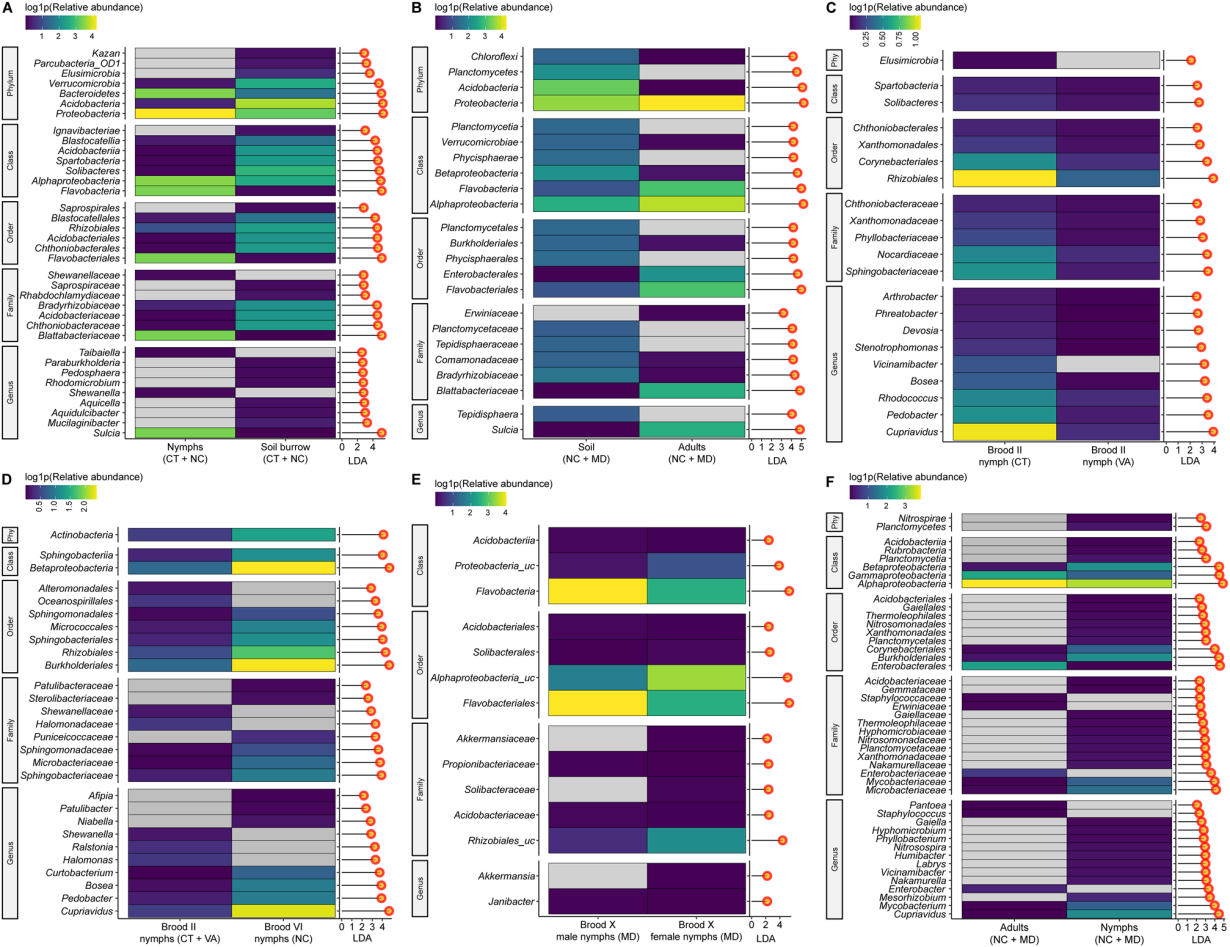
Taxonomic biomarkers calculated using LEfSe^32^. Heatmap (left) provides log10_10_ (relative abundance) of taxonomic biomarkers at the genus level. Bar plot (right) shows linear discriminant analysis (LDA) effect size used to support high-dimensional class comparisons of (**A**) Brood II nymphs (CT) and Brood VI nymphs (NC) versus soil inhabited by nymphs (CT and NC), (**B**) Adults (Brood X, MD, and Brood VI, NC) versus soil (MD and NC), (**C**) Brood II (CT) versus Brood II (VA), (**D**) Brood II nymphs (CT and VA) versus Brood VI nymphs (NC), (**E**) Brood X male nymphs (MD) versus Brood X female nymphs (MD), and (**F**) Adults (Brood X, MD, and Brood VI, NC) versus nymphs (Brood X, MD, and Brood VI, NC).

To evaluate differences among the bacterial communities, taxonomic biomarkers were predicted using linear discriminant analysis (LDA) effect size (LEfSe) method^32^ at differing taxonomic ranking between MTP sets (Fig. 6). Notably, *Sulcia* was a dominant biomarker across taxonomic rankings (*Flavobacteriia, Flavobacteriales*, *Blattabacteriaceae*) between both nymphs (Fig. 6A) and adults (Fig. 6B) and soil samples, detected at significantly higher abundance in cicada gut samples. *Per contra*, *Acidobacteria* was detected as a biomarker between cicadas and soil detected at high abundance in soil. Despite similarities between Brood II populations in CT and VA, a few biomarkers were identified (Fig. 6C), generally detected at higher abundance in the CT population compared to VA. The genera *Cupriavidus*, *Pedobacter*, *Bosea*, and *Curtobacterium* were detected at higher abundance in Brood VI nymphs compared to Brood II nymphs, while a few genera were unique to each brood, namely *Shewanella*, *Ralstonia*, and *Halomonas* exclusive to Brood II and *Afipia*, *Patulibacter*, and *Niabella* exclusive to Brood VI (Fig. 6D). Few biomarkers were detected that consistently differentiated MD Brood X male and female nymphs; however, *Flavobacteria*, detected at increased abundance in males compared to females, was the most significant (Fig. 6E). Biomarkers explaining differences between bacterial communities of adult and nymph cicadas of Broods VI and X were more prevalent (Fig. 6F). For example, the genera *Cupriavidus* and *Mycobacterium* were detected at increased abundance in the nymphs. Members of the classes *Acidobacteriia*, *Rubrobacteria*, and *Planctomycetia* were exclusive to nymphs, and *Betaproteobacteria* were detected at higher abundance in nymphs. In contrast, *Alphaproteobacteria* and *Gammaproteobacteria* were more common to adults.

### Detection of *Hodgkinia* OTUs

**Figure 7:**
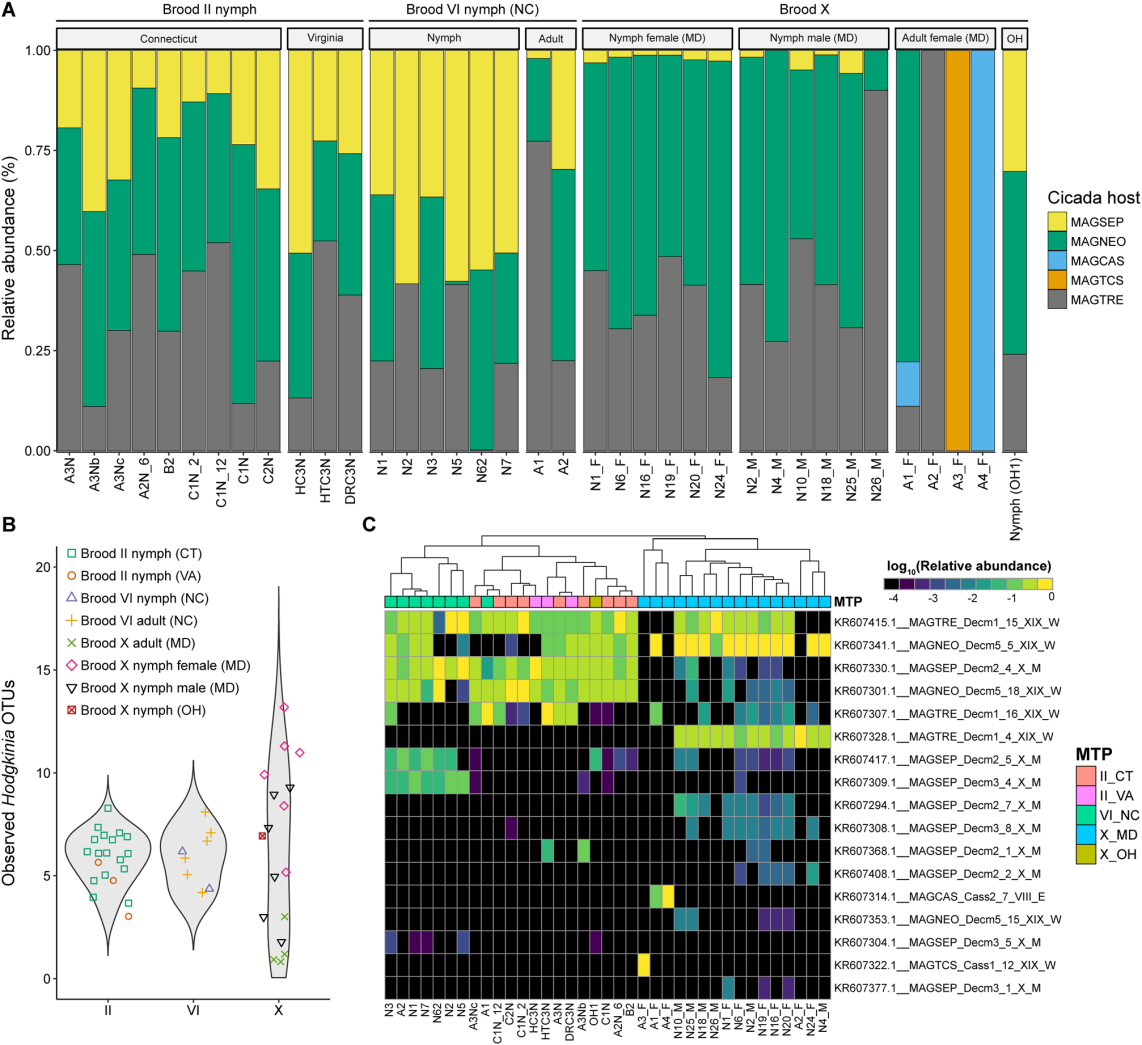
Detection and identification of *Hodgkinia* OTUs. (**A**) Stacked bar plot showing relative abundance of *Hodgkinia* OTUs classified by cicada host species. Characterization of *Hodgkinina* and proposed nomenclature is described elsewhere^33^. MAGNEO, *Magicicada neotredecim*; MAGTRE, *Magicicada tredecim*; MAGCAS, *Magicicada cassini*; MAGTCS, *Magicicada tredecassini*; MAGSEP, *Magicicada septendecim*. (**B**) Violin plot showing number of observed *Hodgkinia* OTUs between brood and MTP set. (**C**) Heatmap showing Log_10_ (relative abundance) of *Hodgkinia* OTUs. Dendrogram shows k-means clustering of samples.

Further taxonomic assignment of unclassified *Alphaproteobacteria* sequencing reads allowed for detection of *Hodgkinia* (Fig. 7). Across all cicadas surveyed, the RA of *Hodgkinia* relative to *Alphaproteobacteria* was variable, ranging from < 1% (N4_M, and MD Brood X adults) to *ca.* 75% (A1). However, *Hodgkinia* RA was observed at highest level in Brood II nymphs (mean = 40.78%, max = 64.19%, min = 26.7%), followed by Brood VI nymphs (mean = 29.43%, max = 38.94%, min = 21.34%), with the lowest RA detected in Brood X nymphs (mean = 10.13%, max = 23.72%, min = < 1%). The RA of reads classified as *Hodgkinia* relative to the total number of reads profiled as *Alphaproteobacteria* in each sample is shown in Fig. S5. *Hodgkinia* OTU previously associated with various *Magicicada *spp.^33^, including *Magicicada neotredecim* (MAGNEO), *Magicicada tredecim* (MAGTRE), *Magicicada cassini* (MAGCAS), *Magicicada tredecassini* (MAGTCS), *Magicicada septendecim* (MAGSEP), were readily detected (Fig. 7A) at higher abundance in nymphs compared to adults (Fig. 7A). In all cicada samples surveyed, with exception of one Brood X nymph (A3_F), multiple copies of the 16S rRNA gene were detected (Fig. 7B), distinguishable as separate OTUs in our genomic data (max = 13). Generally, a greater number of OTUs were observed in nymphs compared to adults. In total, 17 unclassified OTUs were found to be associated with *Hodgkinia*, which were generally differentiated by brood (Fig. 7C).

## Discussion

Insects are both ecologically and economically important, and like all animals, most are known to be associated with microbes such as bacteria and fungi throughout their life cycle that perform key functions^34^. Most studies examining insect–microbe interactions have been focused on one or a few species of obligate endosymbiotic relationships. For example, *Wolbachia* is estimated to be the most abundant of the endosymbiotic bacteria, infecting a range of arthropods and nematodes with varying parasitic and mutualistic associations, from protecting insect hosts from viruses to influencing host fecundity^35,36^. Similarly, *Buchnera* and aphids require each other for survival and reproduction^35^. *Sulcia* and *Hodgkinia* provide essential amino acids to their cicada hosts^6,23–27^, and there is evidence for reduced horizontal gene acquisition among those microbial community members associated with an obligate host^37–39^. It is worth noting that for insects that rely on specialized bacteriome-inhabiting endosymbionts, the gut microbiome has generally been ignored with a few exceptions^24,25,28,29^.

Traditionally, the insect–microbiome interface has proven challenging because many endosymbionts resist genetic manipulation and have not been able to be grown in axenic culture, since many rely on their host and/or other members of the microbial community for metabolic functions and proliferation. Despite these limitations, the “-omics” revolution is accelerating the understanding of host phenotypes and facilitating detection of insect effectors. Table 2 summarizes key studies that use DNA metagenomics and other molecular techniques to explore diversity and function of host-associated microbiomes of various cicada species. These studies show that bacterial communities play crucial roles in nutrition, development, survival, and reproduction of cicadas.

**Table 2:** Microbiota associated with cicadas. Studies are sorted by year of publication.

	Cicada species	Collection date	Collection locality	Study descriptions	Methodology^a^	Important findings and outcomes
Van Leuven et al.^27^	*Tettigades *spp.	2006	Chile	Microbiota in bacteriomes	Genome sequencing and microscopy; SSU rRNA targeted FISH	Speciation events in *Hodgkinia* may have resulted in additional cytologically distinct but metabolically interdependent species in cicadas of the genus *Tettigades*
Campbell et al.^33^	*Magicicada *spp. (Broods VI, VIII, X, XIX, and XXII)	2000–2011	Midwest, Mississippi Valley, and Atlantic regions (USA)	*Hodgkinia* in bacteriomes	Cloning, 16S PCR, and Sanger sequencing	*Hodgkinia* genome has fragmented into multiple new chromosomes or genomes, with some remaining portioned into discrete cells All species examined in *Magicicada* (13-y and 17-y) contain many *Hodgkinia* lineages
Zhou et al.^29^	*Meimuna mongolica*	2011	Shaanxi Province, China	Bacteria in nymphs and adults of both genders	PCR-DGGE and 16S rRNA phylogenetic analysis	Bacteria in the genera *Pseudomonas* and *Enterobacter* were dominant members of gut microflora at all life stages *Pantoea*, *Streptococcus*, and *Uruburuella* were frequent but at lower concentrations PCR-DGGE patterns showed similar patterns across samples indicating *M. mongolica* harbor a characteristic bacterial community independent of developmental stage and gender
Zheng et al.^24^	*Platypleura kaempferi* and *Meimuna mongolica*	2015	Shaanxi Province, China	Bacteria in bacteriomes and organs of reproductive, digestive and excretory systems of two cicada species	RFLP analysis and 16S rRNA sequencing	*Sulcia* was found in the filter chamber and conical segment of both species A novel *Rhizobiales* bacterium showing genetic similarity to *Hodgkinia* was dominant in bacteriomes and ovaries of *P. kaempferi* *Hodgkinia* was not detected in *M. mongolica* suggesting it may have been replaced by another gut microbiota
Łukasik et al.^26^	*Tettigades *spp.	2006–2016	Chile	Microbiota in bacteriomes	Amplicon sequencing (*rpoB*), comparative metagenomics, and microscopy	*Hodgkinia* has repeatedly and independently fractured into complexes of distinct genomic and cellular lineages present in the same host
Wang et al.^25^	*Subpsaltria yangi*	2015–2016	Ningxia Hui Autonomous Region and Hancheng City (China)	Microbiota associated with adults of both sexes (bacteriomes, reproductive organs) and eggs	16S rRNA sequencing combined with primers targeting *Hodgkinia* and FISH	*Sulcia* was observed in all samples A bacterium with *ca.* 80% 16S rDNA similarity to *Hodgkinia* was detected frequently
Cambell et al.^5^	*Diceroprocta semicincta*, *Tettigades* spp., and *Magicicada septendecim* (Broods II, III, IV, V, VI, VII, VIII, IX, X, XIII, XIV, and XXIII)	1981–2017	USA and Chile	Bacteria associated with adult cicadas and eggs	Modeling, FISH, and amplicon sequencing (*Tettigades* spp., *rpoB*; *Magicicada*, *etfD*)	Cicadas increase the titer of *Hodgkinia* cells passed to each egg in response to lineage fragmentation
Matsuura et al.^37^	Multiple spp. (~ 25)	2008–2015	Japan	Microbiota in bacteriomes, ovaries, testes, and whole alimentary tracts	Bacterial 16S rRNA and Fungal 18S rRNA, ITS region, 28S rRNA, RPB1/RPB2, and EF1α sequencing	*Sulcia* is conserved among all cicada species examined, but the majority have lost *Hodgkinia* and instead harbor yeast-like fungal associates
Wang and Wei^30^	*Subpsaltria yangi*	2016–2017	Ningxia Hui Autonomous Region and Hancheng (Shaanxi Province, China)	Bacteria in digestive and excretory organs of two cicada populations with different habitats and diets	16S rRNA sequencing, qPCR, FISH, and RFLP analysis	*Sulcia* distributes in the digestive and excretory glands, in addition to bacteriomes and gonads Core microbiota were observed between the two populations, with most abundant data belonging to *Meiothermus*, *Sulcia*, and *Halomonas*
Huang et al.^7^	*Pycna repanda*	2017–2019	Fengxian County, Shaanxi Province, China	Bacterial communities of salivary glands, bacteriomes, and digestive and reproductive organs	RFLP-based cloning, 16S rRNA sequencing and FISH	Bacterial populations among different gut tissues and bacteriomes of males and females both show similarity but differences were observed in testes and ovaries *Sulcia* and *Hodgkinia* were restricted and dominant in bacteriomes and ovaries Support observations that *Hodgkinia* split into different cellular lineages *Rickettsia* was detected in salivary glands, digestive organs, and testes, whereas *Arsenophonus* was detected in bacteriomes and ovaries Results suggest *Arsenophonus* can coexist with *Sulcia* and *Hodgkinia* within bacteriomes and can be transovarially transmitted
Wang et al.^6^	*Platypleura kaempferi*	2016–2018	Zhouzhi County, Ningshan County, and Yangling District (Shaanxi Province, China)	Bacterial communities in bacteriomes, ovaries, and testes of three representative populations	16S rRNA sequencing combined with light microscopy and confocal imaging	*Sulcia* was detected in all samples with high relative abundance in bacteriomes and ovaries OTUs formerly identified as unclassified *Rhizobiales* were demonstrated to be *Hodgkinia* showing 100% infection rate in all examined samples Cluster analysis revealed significant differences in bacterial communities of the ovaries and testes between locations suggesting microbiota may be influenced by population differentiation of host cicadas and/or host plants of cicadas
Haji et al.^48^	*Kikihia *spp., *Maoricicada *spp., and *Rhodopsalta *spp.	1995–2018	New Zealand	Bacterial communities in gut and reproductive tissues	16S rRNA sequencing combined with qPCR	Gut diversity may be explained by elevational variation across geographic landscape Widespread replacement of obligate bacteria by a domesticated and formerly pathogenic *Ophiocordyceps* fungus was observed

^a^*EF1α* translocation elongation factor 1 alpha, *etfD* electron transfer flavoprotein-ubiquinone oxidoreductase, *FISH* fluorescence in situ hybridization, *ITS* internal transcribed spacer, *PCR* polymerase chain reaction, *PCR-DGGE* polymerase chain reaction denaturing gradient gel electrophoresis, *RPB1* largest subunit of RNA polymerase II, *RPB2* second largest subunit of RNA polymerase II, *qPCR* quantitative, polymerase chain reaction, *rpoB* RNA polymerase subunit beta, *RFLP* restriction fragment length polymorphism, *SSU rRNA* small subunit ribosomal ribonucleic acid.

Synchronous emergence of periodical cicadas coordinates *ca.* 10^6^ to 10^9^ individuals ranging across thousands of hectares^40,41^ presenting a unique opportunity to understand ecological differences, if any, between microbiota associated with *Magicicada* across gender, life stages, and broods. In the study reported here, metagenomic 16S rRNA amplicon sequencing was employed to characterize the gut microbiome components of *Magicicada* cicadas of Broods II, VI, and X collected along the eastern seaboard of the U.S. (Fig. 1).

Various microbial groups are specific in their temperature range preference for growth and survival. Consequently, changes in temperature can have an impact on their microbial community composition. Because periodical cicadas occur from Georgia up through Ohio, Pennsylvania, New York, and Massachusetts along the east coast, differences in their microbial communities could be expected. However, only minor differences were observed in microbial diversity and composition among all broods and species examined (Figs. 2 and 3), with brood X male *M. cassini* nymphs (MD) revealing microbiome differences in beta diversity. However, taxonomic biomarkers with respect to sex of Brood X nymphs were limited (Fig. 6). Other taxonomic biomarkers were detected, namely those differentiating nymph and adult cicadas of broods VI and X. Importantly, this study shows similarities between nymph gut microbiomes between morphologically distinct 17-year *Magicicada M. septendecim* (Broods II and VI) and *M. cassini* (Brood X) and provides evidence of a core microbiome (Figs. 4 and 5). The bacterial phylum *Bacteroidetes* dominated the microbiome of the cicadas examined in this study, with *Actinobacteria*, *Proteobacteria*, *Verrucomicrobia*, and *Acidobacteria* also abundant consistent with prior observations of the cicada microbiomes^24,29^. Similarly, genera were like those previously described^24,29^, namely *Sulcia, Chryseobacterium*, and *Cupriavidus* were most abundant. Endosymbionts belonging to *Flavobacteriaceae*: *Chryseobacterium* have been described in other arthropods, including termites, mosquitoes, cockroaches, ticks, and lice^42–45^, and were detected in MD Brood X *Magicicada* cicadas, though at lower RA in other broods. Bacteria of the genus *Chitinophaga*, shown to be found in association with parasitic fungi^46^, were detected in all samples and at higher abundance in MD Brood X cicadas. Similarly, *Arthrobacter*, suggested to be closely associated with the causative agent of chalky disease, and *Corynebacterium anaganae*, causative agent of a bacterial septicemia of cicadas^47^, were detected and may be significant with respect to overall health of cicadas in MD.

A recent study^6^ employed high-throughput 16S rRNA amplicon sequencing to profile bacterial communities of the cicada *Platypleura kaempferi* (Fabricius) in China. Wang and colleagues^6^ showed that unclassified OTUs, formerly identified as an unclassified *Rhizobiales* bacterium, were in fact the obligate endosymbiont *Hodgkinia*. It is worth noting that we did not detect the *Rhizobiales* bacterium (Genbank accession numbers (KR911840.1, KR911841.1, KR911842.1, and KR911843.1). However, reclassification of reads profiled to *Alphaproteobacteria* (class) but unclassified to lower taxonomic rank proved useful for *Hodgkinia* detection (Fig. 7). We also found that its partner obligate endosymbiont *Sulcia* dominated our gut microbiome samples (Figs. 3E, 5B), supporting results found for cicadas in China and New Zealand^24,48^.

Metagenomic 16S rRNA amplicon sequencing is useful to identify dominant microorganisms present in a biological sample. However, shotgun DNA metagenomics and RNA metatranscriptomics have been shown to detect and identify more genera of bacteria and archaea, along with detection of viruses and eukaryota, compared to 16S rRNA amplicon sequencing^49^. Hence, future metagenomic surveys of periodical cicadas should consider alternative molecular techniques to profile microbial composition to subspecies and determine functional activities encoded by the microbial community. Nonetheless, this study, by applying metagenomic 16S rRNA amplicon sequencing, identified a core microbiome in the whole gut of 17-year periodical cicadas suggesting that the gut microbiome represents an important reservoir of biological diversity. In addition to comparing the gut composition between life stages and brood, this study provides evidence of multiple *Hodgkinia* lineages—supporting previous studies of genome fragmentation and expansion in organelles in other *Magicicada* broods. As nearly every aspect of the cicada microbial community is impacted by its environment, metagenomic analysis of periodical cicadas is useful for evaluating ecological health and the impact of climate variability.

## Methods

### Cicada collection and nucleic acid preparation

Three broods of *Magicicada* cicadas were included in this study: Brood II collected in Northford, Connecticut (10 nymphs; June 2013) and Fredericksburg, Virginia (3 nymphs; June 2013), Brood VI collected in Arden, North Carolina (7 nymphs and 2 adults; May 2017), and Brood X collected in Columbia, MD, 12 nymphs (6 female and 6 male; May 2021) and 4 adults (June 2021). Also included was one Brood X cicada, a nymph ready to emerge four years early, collected in Cincinnati, Ohio (May 2017). All nymphs were pharate, i.e., ready to emerge or had just emerged from the soil and had not yet shed their cuticles. Cicadas were transported to the laboratory and stored at − 20 °C.

Prior to dissection, each cicada was allowed to thaw at room temperature, externally sterilized with 80% (v/v) ethanol or 2% (v/v) bleach for up to 2 min and rinsed with sterilized water. Cicadas were dissected along the dorsal middle line from anus to head with a pair of sterilized scissors and the exoskeleton removed using sterilized fine-tip forceps. Gut tissue (Fig. S2) was separated from other organs, rinsed with normal saline prepared with nuclease free water (0.9% NaCl w/v). Gut tissues were placed in ZymoBIOMICS Lysis Solution (MD Brood X; Zymo Research, Irvine, CA, USA) or Solution CD1 (other cicadas), a lysis buffer included in the DNeasy PowerSoil Kit (Qiagen, Hilden, Germany), at 4 °C until nucleic acid preparation. When DNA could not be prepared immediately, tissue samples were stored at − 20 °C.

MD Brood X tissue samples were homogenized manually in ZymoBIOMICS Lysis Solution using a Teflon tipped pestle tissue grinder (Thomas Scientific, Swedesboro, NJ, USA), and homogenate was transferred to a ZymoBIOMICS Lysis Tube (Zymo Research, Irvine, CA, USA). Tissue samples from other cicadas were placed directly into PowerBead tubes containing solution CD1 (Qiagen, Hilden, Germany). All bead beater tubes were fitted with a 2 mL tube holder assembly and processed at maximum speed for 30 min. Genomic DNA was respectively prepared using either ZymoBIOMICS DNA Miniprep Kit (Zymo Research, Irvine, CA, USA) or DNeasy PowerSoil Kit (Qiagen, Hilden, Germany). DNA was eluted in nuclease free water and stored at − 80 °C. All dissection equipment was sterilized with 10% bleach and treated with UV light for at least one minute prior to dissection and work was done in a laminar flow cabinet.

During nymph collection of Brood II (CT) and Brood VI (NC), soil samples were also collected from the walls of the emergence holes and control samples taken from nearby. DNA was prepared using the DNeasy PowerSoil Kit (Qiagen, Hilden, Germany). Since MD Brood X pharate nymphs were collected after they emerged from the ground, the precise bore holes associated with each nymph could not be identified. To circumvent this issue, we included soil metagenomes from a publicly available 16S (V3–V4) metagenomic survey project (BioProject Accession number PRJNA522438; runs SRR8589945, SRR8589950, and SRR8589955) that was conducted near the study area, during the same months as when cicadas were collected, and with similar soil types, i.e., a suburban lawn. Publicly available metagenomic datasets meeting sufficient standards were not available for VA. Hence, comparisons of VA Brood II nymphs and soil was not done.

### Metagenomic 16S rRNA amplicon sequencing

DNA concentration was measured using the QuantiFluor dsDNA System on a Quantus Fluorometer (Promega, Madison, WI, USA). The 16S rRNA Primers (MD Brood X, V3-V4; Broods II and VI and OH Brood X, V4) within the ribosomal transcript were amplified using the primer pair containing gene‐specific sequences and Illumina adapter overhang nucleotide sequences (Illumina, San Diego, CA, USA). The primer sequences are as follows: V3–V4 (Illumina_F: 5′-CCTACGGGNGGCWGCAG-3′ and Illumina_R: 5′-GACTACHVGGGTATCTAATCC-3′) and V4 (515F: 5′-GTGCCAGCMGCCGCGGTAA-3′, and 806R: 5′-GGACTACHVGGGTWTCTAAT-3′).

Amplicon PCR was performed to amplify the template from input of DNA samples. Briefly, each PCR reaction (25 μL) contained 12.5 ng sample DNA as input, 12.5 μL 2 × KAPA HiFi HotStart ReadyMix (Kapa Biosystems, Wilmington, MA), and 5 μL of 1 μM of each primer. PCR reactions were carried out using the following protocols: initial denaturation (95 °C for 3 min); 25 cycles (V3–V4) or 35 cycles (V4) of denaturation (95 °C, 30 s), annealing (55 °C, 30 s or 60 s), and extension (72 °C, 30 s or 60 s); and a final elongation (72 °C, 5 min). The resulting PCR product was cleaned to eliminate excess nucleotides (< 100 bp), residual primers, and nonspecific PCR products employing Mag-Bind RxnPure Plus magnetic beads (Omega Bio-tek, Norcross, GA) or AMPure XP beads (Beckman Coulter, Indianapolis, IN), per manufacturer’s specifications. For V3–V4 sample reactions, a second index PCR amplification to incorporate barcodes and sequencing adapters into final PCR product was performed in 25 μL reactions, using previously mentioned master mix and thermocycler conditions, with exception to the number of cycling steps which was reduced to eight cycles—totaling 33 cycles across both PCR reactions. Individual sample libraries were normalized with the Mag-Bind EquiPure Library Normalization Kit (Omega Bio-tek, Norcross, GA) and pooled. Pooled libraries were validated with Agilent 2200 TapeStation and sequenced (2 × 300 bp paired read setting) on the MiSeq (Illumina, San Diego, CA). For technical control, the wash fluid was profiled from the OH Brood X nymph (Fig. S6). In addition, a no template control (NTC), consisting of nuclease free water, and sequencing standard, i.e., ZymoBIOMICS™ Microbial Community Standard (Zymo Research, Irvine, CA, USA), were included for quality control.

### Metagenomic taxonomic profiling

We initially sought to identify potentially unique bacterial populations residing in different gut organs of CT Brood II nymphs, including filter chamber (n = 1), midgut (n = 4), Malpighian tubules and hindgut (n = 2), and rectum (n = 1). However, metagenomic 16S rRNA amplicon sequencing analysis yielded only limited statistical differences between the midgut and other organs (Fig. S7). Hence, subsequent analysis of CT Brood II nymphs (and analysis of other cicada samples) was done by pooling sequencing reads from like samples and normalizing the size of pooled libraries to 100,000 sequencing reads. In total, 39 cicadas (33 nymphs and 6 adults) were prepared for the following experiments.

For paired-end sequencing, sequences representing the same PCR amplicon, i.e., forward and reverse reads, were merged and the resulting overlapped sequence extracted using VSEARCH v2.21.1^50^. Primer sequences were trimmed, and sequences were quality filtered to remove low quality reads and singletons. Reads not predicted to be of 16S rRNA origin were discarded. The VSEARCH program^50^ was used to search the EzBioCloud 16S database vPKSSU4.0^51^ and calculate sequence similarities between reads. A cutoff of 97% similarity was used to define 16S rRNA OTUs for species-level identification. Sequences not matched by 97% percent were clustered using the UCLUST algorithm^52^, with a 97% similarity boundary, and an OTU was defined as a group of clusters. Other sequence similarity cutoffs used for higher taxonomic ranks: genus [94.5%, 97%), family [86.5%, 94.5%), order [82%, 86.5%), class [78.5%, 82%), and phylum [75%, 78.5%). Cutoff values have been described previously^53^. Using OTU information, i.e., the number of OTUs and sequences profiled in each OTU, taxa RA, and various measures of diversity were calculated. Reads classified to *Alphaproteobacteria* (class) but unclassified to lower taxonomic rank were subjected to further analysis for *Hodgkinia* detection; an observation proposed by Wang and colleagues^6^.

### *Hodgkinia* detection

Detection of *Hodgkinia* genome via short-insert Illumina sequencing is complex, often requiring alternative sequencing methods or additional analyses^6,26,27,33^. Using the search term ‘*Hodgkinia* 16S rRNA’ on the NCBI nucleotide database with a sequence length filter set to between 500 and 1500 bp yielded 267 *Hodgkinia* isolates obtained from various *Magicicada* spp., as of July 27, 2022. The 267 *Hodgkinia* 16S sequences were downloaded and curated using the Cluster Database at High Identity with Tolerance (CD-HIT-EST) tool v4.8.1^54^ with ANI threshold of 97%, i.e., the same similarity score used for OTU identification during metagenomic taxonomic profiling. Reference databases were built from the resulting in 82 clusters.

To avoid the misidentification of bacterial species owing to short sequence reads or those with high similarity, a two-step read mapping method was employed, an approach established elsewhere^55^. First, the BBMap (BBTools) algorithm v38.90^56^ was used to align unclassified *Alphaproteobacteria* reads against the curated *Hodgkinia* reference database. Sequence similarity for each read mapping to reference sequence was calculated, and reads at least 97% identical to the reference sequence were extracted and re-aligned against the reference database using the Basic Local Alignment Search Tool for nucleotide query (BLASTN) v2.12^57^. Reads passing the filtering criteria (E-value < 10E−20, percent identity > 99, length > 200, and query coverage per high-score segment pair ≥ 99) were reclassified under the corresponding *Hodgkinia* OTU for subsequent analysis.

### Statistical analysis

Employing metagenomic 16S rRNA amplicon sequencing, we sought to address the testable hypotheses detailed in Table 1. Copy number correction for the 16S rRNA gene was applied prior to comparative analysis using the PICRUSt2 v2.4.2^58^ to generate normalized 16S copy numbers for all species/phylotypes in the EzBioCloud 16S database^51^. The necessity of 16S copy number correction is detailed elsewhere^59–61^.

Measures of alpha diversity, including species richness (ACE, Chao1, Jackknife, and the number of OTUs in each sample) and diversity indices (Shannon, NP Shannon, Simpson, and overall phylogenetic diversity), were calculated using the EzBioCloud comparative analyzer for MTP sets^51^. Briefly, Shannon entropy of counts was calculated based on the description given in the Species Diversity and Richness manual^62^. However, log base 2 was used as default instead of the natural logarithm (log_e_). Simpson’s index was defined as 1 − *Dominance*, described previously^63^. Richness was compared between groups using the Wilcoxon rank-sum test^64^. Good’s coverage index^65^ was used to measure sample completeness, i.e., the proportion of total number of reads in a sample library belonging to OTUs represented in the sample.

Random sampling with replacement was used to normalize sequencing depth, and the RA of bacterial taxa in each sample was used for NMDS, employing Bray–Curtis distance measure^66^. PERMANOVA was calculated using Adonis^67^ on the Bray–Curtis Beta diversity distance measure and corrected for the rate of false discovery. Taxonomic biomarkers were predicted using LEfSe method to support high-dimensional class comparisons^32^, and biomarkers were found between MTP sets using a P value < 0.05 (Kruskal–Wallis test) and an LDA score (log_10_) > 2.

## Supplementary Information

Dataset S1.

Supplementary Figures.

## Data Availability

Sequencing data generated for all samples in this study have been deposited in public repositories. Brood X (OH 2017 and MD 2021) sequencing data have been deposited in the National Center for Biotechnology Information (NCBI) Sequence Read Archive database under BioProject PRJNA825527. Brood II (CT and VA, 2013) sequencing data have been deposited in the Metagenomics Rapid Annotation using the Subsystem Technology (MG-RAST) server under Study ID mgp104267. Individual accession numbers are listed in supplementary Dataset S1.

## References

[1] Panthee B, Gyawali S, Panthee P, Techato K (2022). Environmental and human microbiome for health. Life.

[2] Eleftherianos I, Atri J, Accetta J, Castillo J (2013). Endosymbiotic bacteria in insects: Guardians of the immune system?. Front. Physiol..

[3] Kikuchi Y (2009). Endosymbiotic bacteria in insects: Their diversity and culturability. Microbes Environ..

[4] Bennett GM, Moran NA (2015). Heritable symbiosis: The advantages and perils of an evolutionary rabbit hole. Proc. Natl. Acad. Sci..

[5] Campbell MA (2018). Changes in endosymbiont complexity drive host-level compensatory adaptations in cicadas. MBio.

[6] Wang D, Liu Y, Su Y, Wei C (2021). Bacterial communities in bacteriomes, ovaries and testes of three geographical populations of a sap-feeding insect, *Platypleura*
*kaempferi* (Hemiptera: Cicadidae). Curr. Microbiol..

[7] Huang Z, Wang D, Li J, Wei C, He H (2020). Transovarial transmission of bacteriome-associated symbionts in the cicada *Pycna repanda* (Hemiptera: Cicadidae). Appl. Environ. Microbiol..

[8] Sanborn A (2013). Catalogue of the Cicadoidea (Hemiptera: Auchenorrhyncha).

[9] Karban R (2014). Transient habitats limit development time for periodical cicadas. Ecology.

[10] Williams KS, Simon C (1995). The ecology, behavior, and evolution of periodical cicadas. Annu. Rev. Entomol..

[11] Teiji S (2013). Independent divergence of 13- and 17-y life cycles among three periodical cicada lineages. Proc. Natl. Acad. Sci..

[12] Simon C, Cooley JR, Karban R, Sota T (2022). Advances in the evolution and ecology of 13- and 17-year periodical cicadas. Annu. Rev. Entomol..

[13] Fujisawa T (2018). Triplicate parallel life cycle divergence despite gene flow in periodical cicadas. Commun. Biol..

[14] Alexander, R. D. & Moore, T. E. The evolutionary relationships of 17-year and 13-year cicadas, and three new species (Homoptera, Cicadidae, Magicicada) (1962).

[15] Cooley JR (2015). The distribution of periodical Cicada (Hemiptera: Cicadidae: Magicicada) Brood II in 2013: Disjunct emergences suggest complex brood origins. Am. Entomol..

[16] Cooley JR, Marshall DC, Simon C (2021). Documenting single-generation range shifts of periodical Cicada Brood VI (Hemiptera: Cicadidae: *Magicicada* spp.). Ann. Entomol. Soc. Am..

[17] Kritsky G (2021). One for the books: The 2021 emergence of the periodical Cicada Brood X. Am. Entomol..

[18] Koyama T (2016). Genomic divergence and lack of introgressive hybridization between two 13-year periodical cicadas support life cycle switching in the face of climate change. Mol. Ecol..

[19] Marshall DC, Cooley JR (2000). Reproductive character displacement and speciation in periodical cicadas, with description of new species, 13-year Magicicada neotredecem. Evol. Int. J. Org. Evol..

[20] Kritsky, G., Webb, J., Folsom, M. & Pfiester, M. Observations of Periodical Cicadas (BroodX) in Indiana and Ohio in 2004 (Hemiptera: Cicadidae: Magicicada spp.). In *Proceedings of the Indiana Academy of Science* vol. 114 65–69 (2005).

[21] White J, Strehl CE (1978). Xylem feeding by periodical cicada nymphs on tree roots. Ecol. Entomol..

[22] Douglas A (2006). Phloem-sap feeding by animals: Problems and solutions. J. Exp. Bot..

[23] McCutcheon JP, McDonald BR, Moran NA (2009). Convergent evolution of metabolic roles in bacterial co-symbionts of insects. Proc. Natl. Acad. Sci..

[24] Zheng Z, Wang D, He H, Wei C (2017). Bacterial diversity of bacteriomes and organs of reproductive, digestive and excretory systems in two cicada species (Hemiptera: Cicadidae). PLoS One.

[25] Wang D, Huang Z, He H, Wei C (2018). Comparative analysis of microbial communities associated with bacteriomes, reproductive organs and eggs of the cicada *Subpsaltria yangi*. Arch. Microbiol..

[26] Łukasik P (2018). Multiple origins of interdependent endosymbiotic complexes in a genus of cicadas. Proc. Natl. Acad. Sci..

[27] Van Leuven JT, Meister RC, Simon C, McCutcheon JP (2014). Sympatric speciation in a bacterial endosymbiont results in two genomes with the functionality of one. Cell.

[28] Zhong H, Wei C, Zhang Y (2013). Gross morphology and ultrastructure of salivary glands of the mute cicada *Karenia caelatata* Distant (Hemiptera: Cicadoidea). Micron.

[29] Zhou W, Nan X, Zheng Z, Wei C, He H (2015). Analysis of inter-individual bacterial variation in gut of cicada *Meimuna mongolica* (Hemiptera: Cicadidae). J. Insect Sci..

[30] Wang D, Wei C (2020). Bacterial communities in digestive and excretory organs of cicadas. Arch. Microbiol..

[31] Boyce GR (2019). Psychoactive plant- and mushroom-associated alkaloids from two behavior modifying cicada pathogens. Fungal Ecol..

[32] Segata N (2011). Metagenomic biomarker discovery and explanation. Genome Biol..

[33] Campbell MA (2015). Genome expansion via lineage splitting and genome reduction in the cicada endosymbiont “Hodgkinia”. Proc. Natl. Acad. Sci..

[34] Gupta, A. & Nair, S. Dynamics of insect–microbiome interaction influence host and microbial symbiont. *Front. Microbiol*. **11** (2020). 10.3389/fmicb.2020.01357 PMC7333248 32676060

[35] Smith TE, Moran NA (2020). Coordination of host and symbiont gene expression reveals a metabolic tug-of-war between aphids and Buchnera. Proc. Natl. Acad. Sci..

[36] Taylor MJ, Bordenstein SR, Slatko B (2018). Microbe Profile: Wolbachia: A sex selector, a viral protector and a target to treat filarial nematodes. Microbiol. Read. Engl..

[37] Matsuura Y (2018). Recurrent symbiont recruitment from fungal parasites in cicadas. Proc. Natl. Acad. Sci..

[38] Moran NA (1996). Accelerated evolution and Muller’s rachet in endosymbiotic bacteria. Proc. Natl. Acad. Sci..

[39] Mira A, Ochman H, Moran NA (2001). Deletional bias and the evolution of bacterial genomes. Trends Genet..

[40] Nowlin WH (2007). Allochthonous subsidy of periodical cicadas affects the dynamics and stability of pond communities. Ecology.

[41] Whiles MR, Callaham MA, Meyer CK, Brock BL, Charlton RE (2001). Emergence of periodical cicadas (*Magicicada cassini*) from a Kansas riparian forest: Densities, biomass and nitrogen flux. Am. Midl. Nat..

[42] Doña, J., Virrueta Herrera, S., Nyman, T., Kunnasranta, M. & Johnson, K. P. Patterns of microbiome variation among infrapopulations of permanent bloodsucking parasites. *Front. Microbiol.* **12** (2021). 10.3389/fmicb.2021.642543 PMC8085356 33935998

[43] Burešová V, Franta Z, Kopáček P (2006). A comparison of *Chryseobacterium indologenes* pathogenicity to the soft tick *Ornithodoros moubata* and hard tick *Ixodes*
*ricinus*. J. Invertebr. Pathol..

[44] Montasser AA (2005). Gram-negative bacteria from the camel tick *Hyalomma dromedarii* (Ixodidae) and the chicken tick *Argas persicus* (Argasidae) and their antibiotic sensitivities. J. Egypt. Soc. Parasitol..

[45] Campbell CL, Mummey DL, Schmidtmann ET, Wilson WC (2004). Culture-independent analysis of midgut microbiota in the arbovirus vector *Culicoides sonorensis* (Diptera: Ceratopogonidae). J. Med. Entomol..

[46] Huang, A. *et al.* Analysis of internal and external microorganism community of wild cicada flowers and identification of the predominant *Cordyceps cicadae* fungus. *Front. Microbiol.* **12** (2021). 10.3389/fmicb.2021.752791 PMC8656164 34899639

[47] Lüthy P, Soper RS (1969). Chalky disease, a bacterial septicemia of the cicada *Okanagana rimosa* Say. J. Invertebr. Pathol..

[48] Haji D (2021). Host-associated microbial diversity in New Zealand cicadas uncovers elevational structure and replacement of obligate bacterial endosymbionts by *Ophiocordyceps* fungal pathogens. bioRxiv..

[49] Brumfield KD, Huq A, Colwell RR, Olds JL, Leddy MB (2020). Microbial resolution of whole genome shotgun and 16S amplicon metagenomic sequencing using publicly available NEON data. PLoS One.

[50] Rognes T, Flouri T, Nichols B, Quince C, Mahé F (2016). VSEARCH: A versatile open source tool for metagenomics. PeerJ.

[51] Yoon S-H (2017). Introducing EzBioCloud: A taxonomically united database of 16S rRNA gene sequences and whole-genome assemblies. Int. J. Syst. Evol. Microbiol..

[52] Edgar RC (2010). Search and clustering orders of magnitude faster than BLAST. Bioinformatics.

[53] Yarza P (2014). Uniting the classification of cultured and uncultured bacteria and archaea using 16S rRNA gene sequences. Nat. Rev. Microbiol..

[54] Fu L, Niu B, Zhu Z, Wu S, Li W (2012). CD-HIT: Accelerated for clustering the next-generation sequencing data. Bioinformatics (Oxf., Engl.).

[55] Miao J (2017). 16SPIP: A comprehensive analysis pipeline for rapid pathogen detection in clinical samples based on 16S metagenomic sequencing. BMC Bioinform..

[56] Joint Genome Institute. BBMap short read aligner, and other bioinformatics tools (2020).

[57] Camacho C (2009). BLAST+: Architecture and applications. BMC Bioinform..

[58] Langille MGI (2013). Predictive functional profiling of microbial communities using 16S rRNA marker gene sequences. Nat. Biotechnol..

[59] Kembel SW, Wu M, Eisen JA, Green JL (2012). Incorporating 16S gene copy number information improves estimates of microbial diversity and abundance. PLoS Comput. Biol..

[60] Angly FE (2014). CopyRighter: A rapid tool for improving the accuracy of microbial community profiles through lineage-specific gene copy number correction. Microbiome.

[61] Vandeputte D (2017). Quantitative microbiome profiling links gut community variation to microbial load. Nature.

[62] Seaby RM, Henderson PA (2006). Species Diversity and Richness.

[63] Hammer, O. Diversity. https://web.archive.org/web/20200217074057/http://folk.uio.no/ohammer/past/diversity.html (2003).

[64] Wilcoxon F (1945). Individual comparisons by ranking methods. Biom. Bull..

[65] Good IJ (1953). The population frequencies of species and the estimation of population parameters. Biometrika.

[66] Bray JR, Curtis JT (1957). An ordination of the upland forest communities of Southern Wisconsin. Ecol. Monogr..

[67] Anderson MJ (2001). A new method for non-parametric multivariate analysis of variance. Austral Ecol..

[68] Environmental Systems Research Institute. ARCgis Online (2022).

