# Supplementary material for: Gut microbiome insights from 16S rRNA analysis of 17-year periodical cicadas (Hemiptera: Magicicada spp.) Broods II, VI, and X
Source: Sci Rep. 2022 Oct 10;12:16967. doi: 10.1038/s41598-022-20527-7 (PMC9550851; doi:10.1038/s41598-022-20527-7)
Supplement: Supplementary file 2 — Supplementary Figures. [file 41598_2022_20527_MOESM2_ESM.pdf]

Gut microbiome insights from 16S rRNA analysis of 17-year periodical cicadas (*Hemiptera*: *Magicicada spp.*) Broods II, VI, and X

Kyle D. Brumfield[1,2], Michael J. Raupp[3], Diler Haji[4], Chris Simon[4], Joerg Graf[5], John R. Cooley[6], Susan T. Janton[5], Russell C. Meister[4], Anwar Huq[1], Rita R. Colwell[1,2,*], Nur A. Hasan[7,*]

[1] Maryland Pathogen Research Institute, University of Maryland, College Park, MD, 20742, USA

[2] University of Maryland Institute for Advanced Computer Studies, University of Maryland, 20742, College Park, MD, USA

[3] Department of Entomology, University of Maryland, College Park, MD, 20742, USA

[4] Department of Ecology and Evolutionary Biology, University of Connecticut, Storrs, CT, 06269, USA

[5] Department of Molecular and Cell Biology, University of Connecticut, Storrs, CT, 06269, USA

[6] Department of Ecology and Evolutionary Biology, University of Connecticut, Hartford, CT, 06103, USA

[7] EZbiome Inc., Gaithersburg, MD, 20878, USA

*Correspondence: RRC, rcolwell@umd.edu; NAH, hasan@ezbiome.com

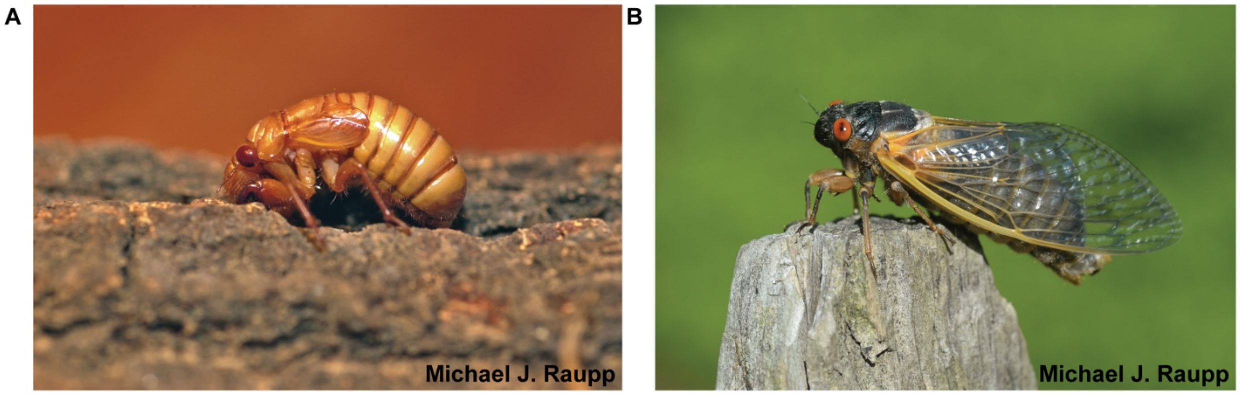

Figure S1: Photographs of A) nymph and B) adult 17-year periodical cicadas (*Magicicada spp.* Brood X).

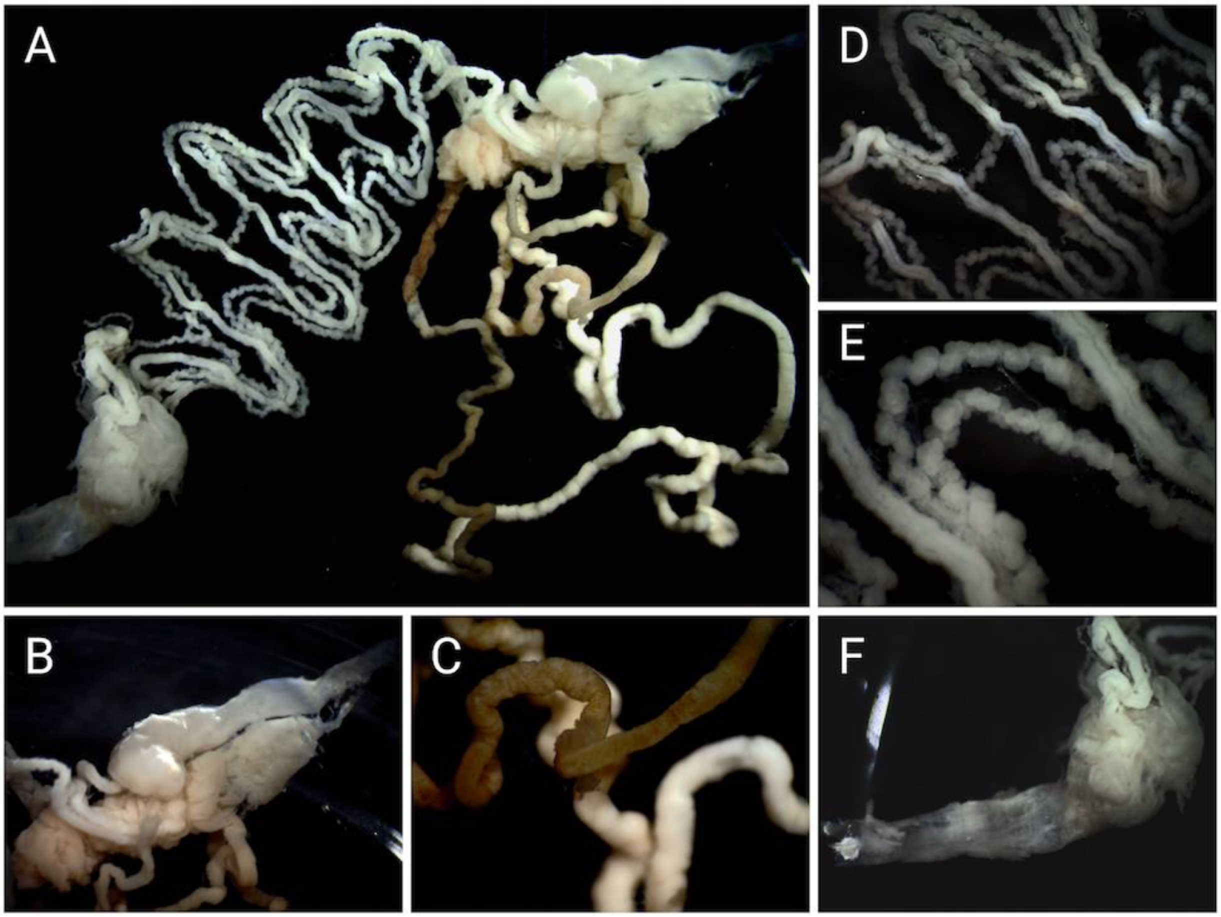

Figure S2: Image of gut tissue dissected from nymphal *Magicicada septendecium*. Shown are **A)** Complete gut, **B)** Filter chamber, **C)** Midgut, **D-E)** Malpighian tubules and hindgut, and **F)** Rectum. Adult gut anatomy and other *Magicicada spp*., appears identical to that of the *M. septendecium* nymph.

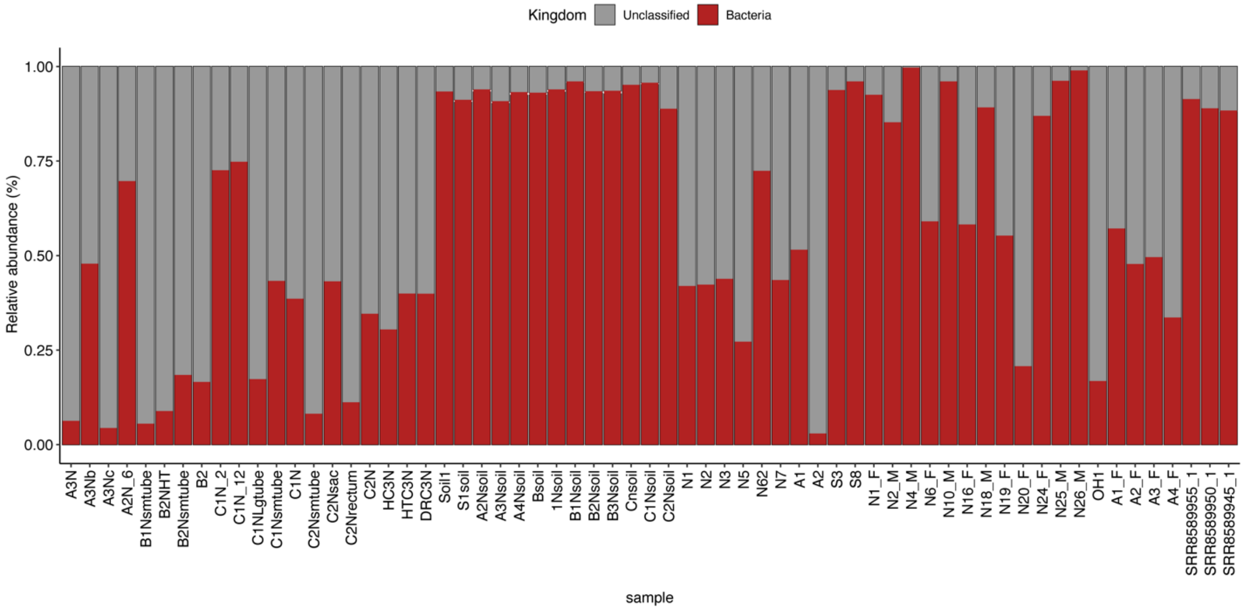

Figure S3: Stacked bar plot showing relative abundance of reads classified as bacteria relative to the total number of reads generated in each sample.

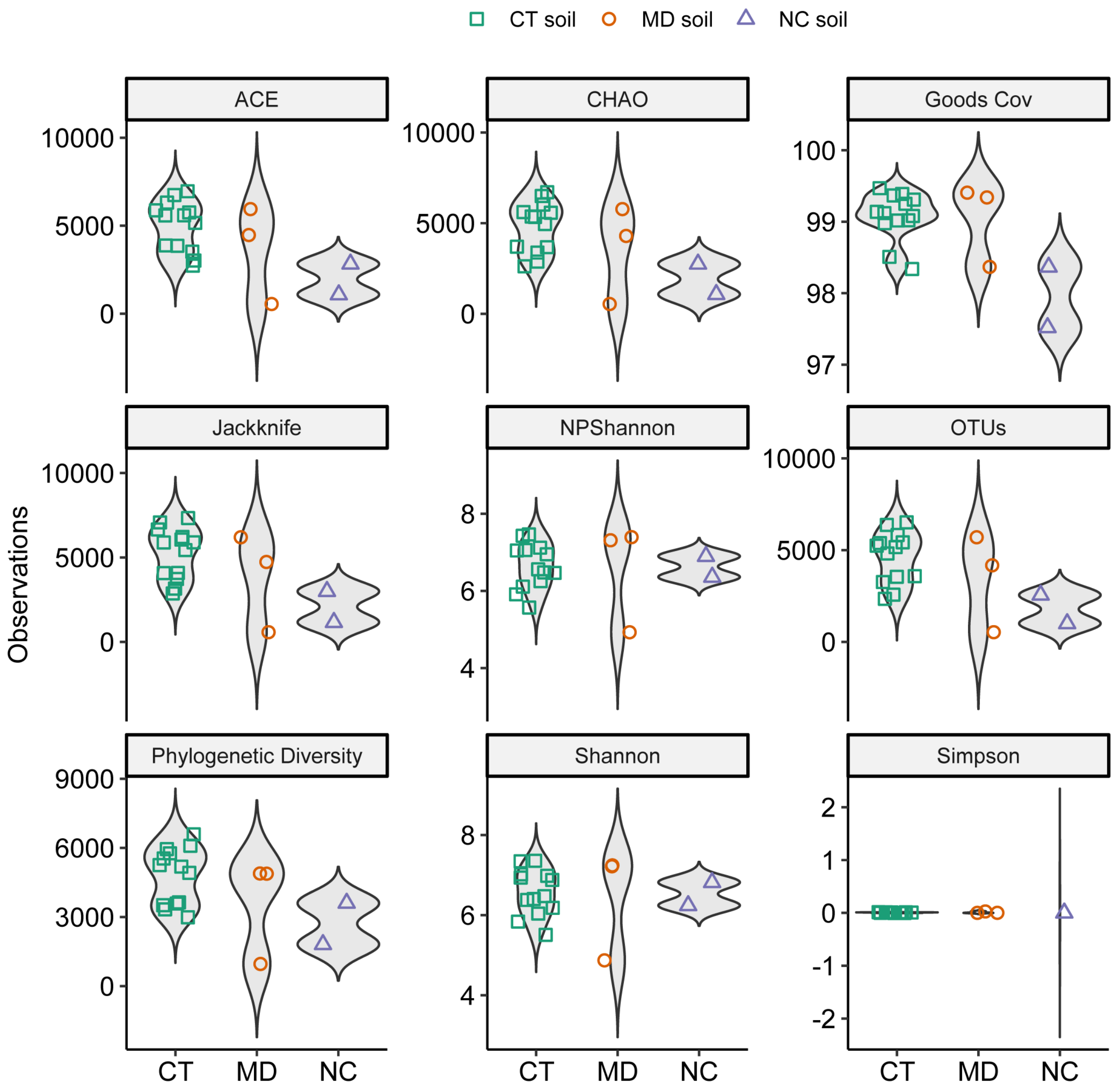

Figure S4: Violin plots showing alpha diversity comparison between MTP sets of soil samples.

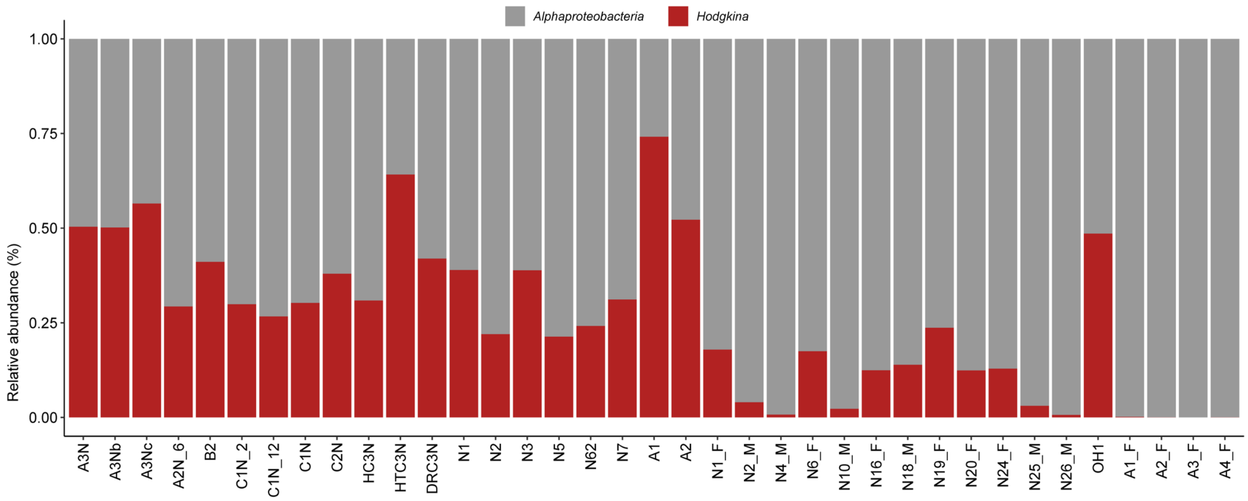

Figure S5: Stacked bar plot showing relative abundance of reads classified as *Hodgkinia* relative to the total number of reads profiled as *Alphaproteobacteria* in each sample.

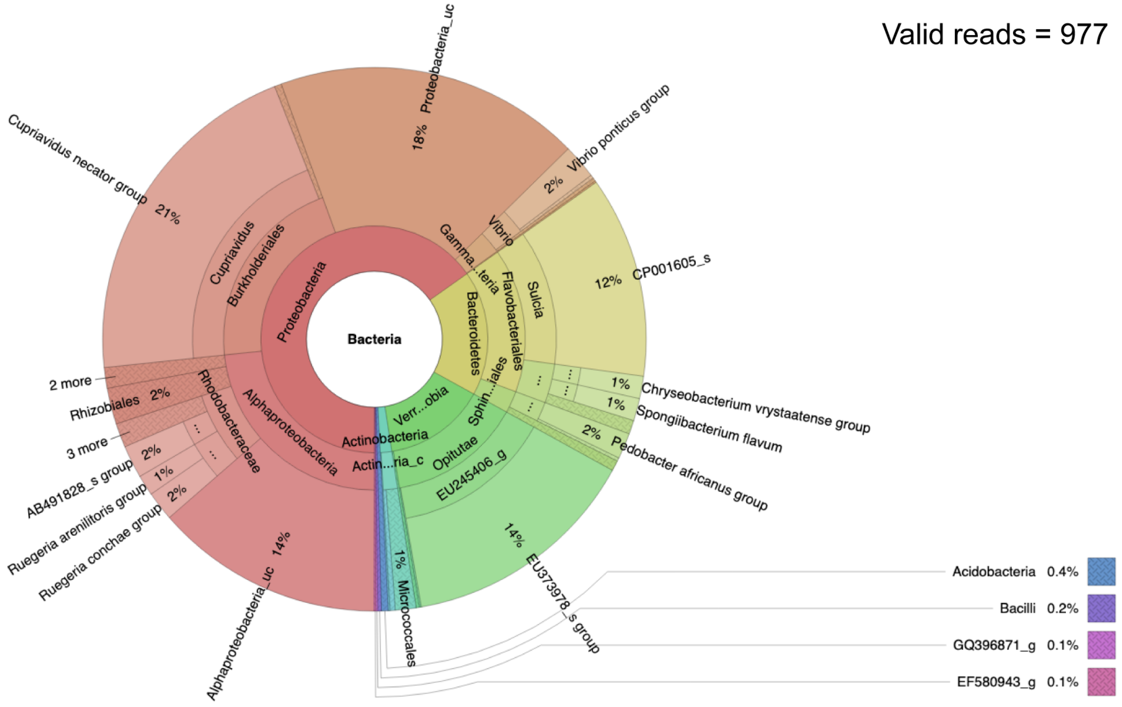

Figure S6: Krona plot showing relative abundance of bacteria profiled in the wash fluid of the OH Brood X nymph.

Phylum

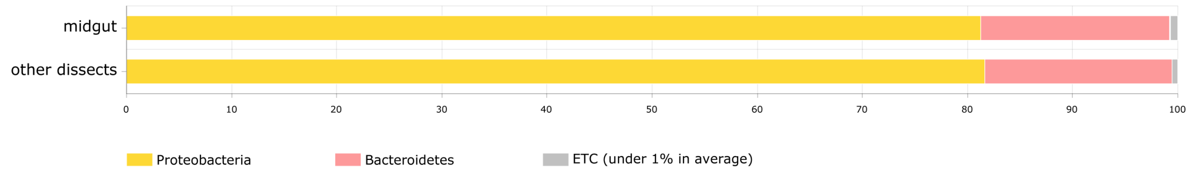

Order

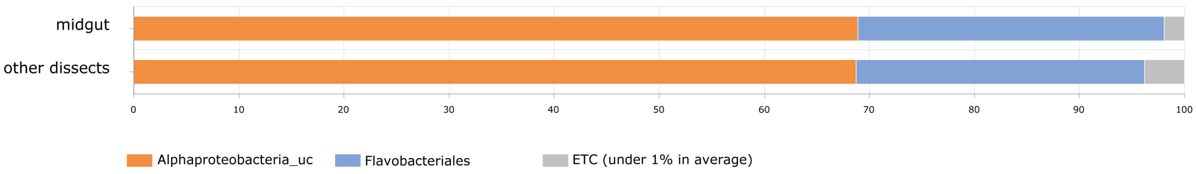

PERMANOVA results

All sets

p-value<br>N.S. (p=0.774)	Sets<br>2
MTPs<br>8	No. of permutations<br>999

Pair-wise

		pseudo-F	p-value	q-value
midgut	other dissects	0.067	0.774	0.774

Inter-set distances

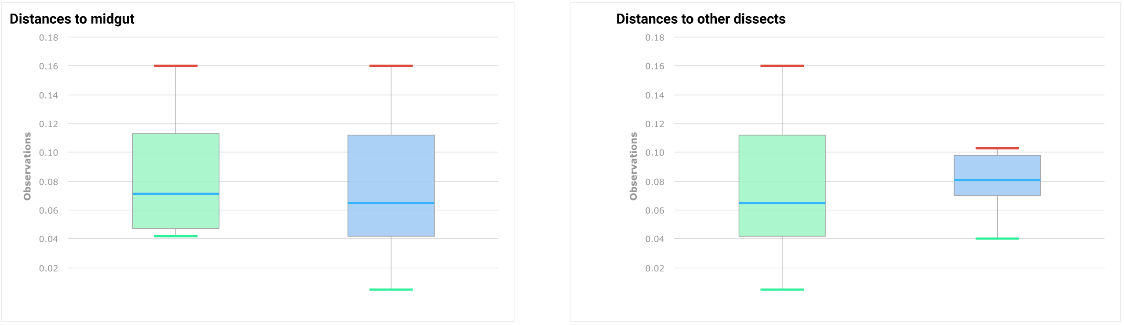

LEfSe analysis

Taxon name	Taxon rank	Taxonomy	LDA effect size	p-value	p-value (FDR)	Max group	midgut	other dissects
Tenericutes	Phylum	Bacteria	2.20018	0.04722	0.42845	hindgut	0.02907	0.00000
Ralstonia pickettii group	Species	Bacteria: Proteobacteria: Betaproteobacteria: Burkholderiales: Ralstonia_f: Ralstonia	2.19196	0.04722	0.42845	other dissects	0.00000	0.03489

Figure S7: Comparison of bacterial populations residing in different gut organs of CT Brood II nymphs.
